# Fgf-driven Tbx protein activities directly induce *myf5* and *myod* to initiate zebrafish myogenesis

**DOI:** 10.1242/dev.184689

**Published:** 2020-04-28

**Authors:** Daniel P. S. Osborn, Kuoyu Li, Stephen J. Cutty, Andrew C. Nelson, Fiona C. Wardle, Yaniv Hinits, Simon M. Hughes

**Affiliations:** Randall Centre for Cell and Molecular Biophysics, New Hunt's House, Guy's Campus, King's College London, SE1 1UL, UK

**Keywords:** Muscle, Zebrafish, Myosin, Myod, Myf5, Myogenin, Hedgehog, Fgf, Spt, Tbx16, Ntl, Tbxta

## Abstract

Skeletal muscle derives from dorsal mesoderm formed during vertebrate gastrulation. Fibroblast growth factor (Fgf) signalling cooperates with Tbx transcription factors to promote dorsal mesoderm formation, but their role in myogenesis has been unclear. Using zebrafish, we show that dorsally derived Fgf signals act through Tbx16 and Tbxta to induce slow and fast trunk muscle precursors at distinct dorsoventral positions. Tbx16 binds to and directly activates the *myf5* and *myod* genes, which are required for commitment to myogenesis. Tbx16 activity depends on Fgf signalling from the organiser. In contrast, Tbxta is not required for *myf5* expression, but binds a specific site upstream of *myod* that is not bound by Tbx16 and drives (dependent on Fgf signals) *myod* expression in adaxial slow precursors, thereby initiating trunk myogenesis. After gastrulation, when similar muscle cell populations in the post-anal tail are generated from tailbud, declining Fgf signalling is less effective at initiating adaxial myogenesis, which is instead initiated by Hedgehog signalling from the notochord. Our findings suggest a hypothesis for ancestral vertebrate trunk myogenic patterning and how it was co-opted during tail evolution to generate similar muscle by new mechanisms.

This article has an associated ‘The people behind the papers’ interview.

## Abstract (precis)

**Highlighted Article:** Tbx16 and Tbxta activate *myf5* and *myod* directly during the earliest myogenesis in zebrafish, and Fgf signalling acts through Tbx16 to drive myogenesis in trunk but not tail.

## INTRODUCTION

Sarcomeric muscle arose early in animal evolution and is today regulated by similar gene families in *Drosophila* and vertebrates e.g. *mef2* genes (Taylor and Hughes, 2017). Skeletal myogenesis is initiated during gastrulation. Yet conserved bilaterian pathways leading specifically to skeletal (as opposed to cardiac or visceral) muscle have been hard to discern, perhaps because new regulatory steps have evolved in each phylum since their divergence.

A key step in vertebrate evolution was the chordate transition through which animals acquired notochord, post-anal tail, gill slits and dorsal neural tube, facilitating swimming (Brunet et al., 2015; Gee, 2018; Gerhart, 2001; Satoh et al., 2012). Throughout vertebrates, the notochord patterns the neural tube and paraxial mesodermal tissue by secreting Hedgehog (Hh) signals that promote motoneuron and early muscle formation (Beattie et al., 1997; Blagden et al., 1997; Du et al., 1997; Münsterberg et al., 1995; Roelink et al., 1994). Nevertheless, in the absence of either notochord or Hh signalling, muscle is formed in vertebrate somites (Blagden et al., 1997; Dietrich et al., 1999; Du et al., 1997; Grimaldi et al., 2004; Zhang et al., 2001). How might deuterostome muscle have formed prior to evolution of the notochord?

A change in function of the *Tbxt* gene, a T-box (Tbx) family paralogue, occurred during chordate evolution such that *Tbxt* now directly controls formation of posterior mesoderm, notochord and post-anal tail in vertebrates (Chiba et al., 2009; Showell et al., 2004). Hitherto, *Tbxt* may have distinguished ectoderm from endoderm and regulated formation of the most posterior mesendoderm (Arenas-Mena, 2013; Kispert et al., 1994; Woollard and Hodgkin, 2000; Yasuoka et al., 2016). In zebrafish, Tbxt genes also promote slow myogenesis (Coutelle et al., 2001; Halpern et al., 1993; Martin and Kimelman, 2008; Weinberg et al., 1996). Other Tbx genes, such as *Tbx1*, *Tbx4*, *Tbx5*, *Tbx16* and *Tbx6*, also influence sarcomeric muscle development in both fish and amniotes (Chapman and Papaioannou, 1998; Griffin et al., 1998; Hasson et al., 2010; Kimmel et al., 1989; Manning and Kimelman, 2015; Nandkishore et al., 2018; Weinberg et al., 1996; Windner et al., 2015). For example, the Tbx6 family is implicated in early stages of paraxial mesoderm commitment, somite patterning and myogenesis (Bouldin et al., 2015; Chapman and Papaioannou, 1998; Kimmel et al., 1989; Manning and Kimelman, 2015; Nikaido et al., 2002; White et al., 2003; Windner et al., 2012). It is unclear, however, whether the Tbx genes promote myogenesis directly, and/or are required for earlier events in mesoderm development that are necessary for myogenesis.

**Fig. 1.**
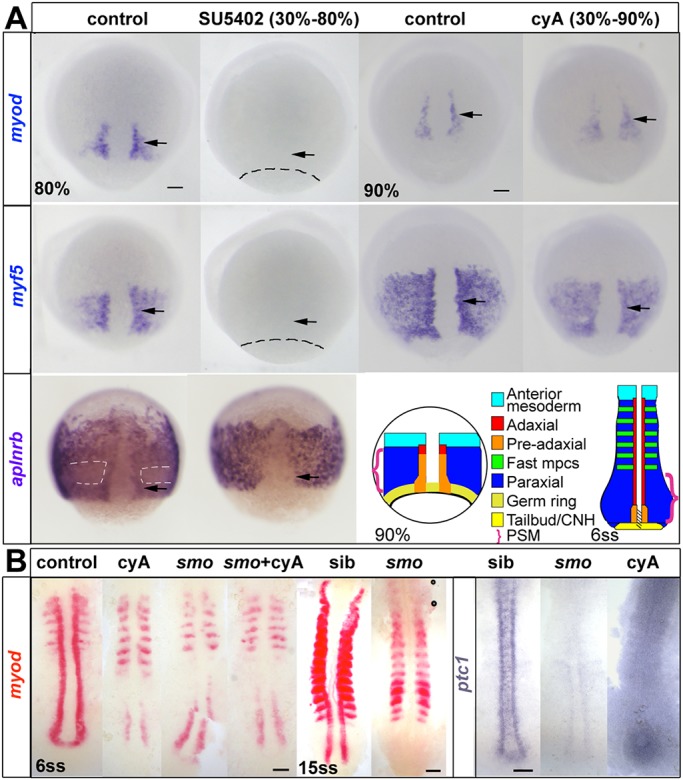
**Inhibition of Fgf signalling blocks initial induction of *myod* and *myf5* expression.** ISH for *myod* and *myf5* in control untreated, cyA-treated (100 µM) and SU5402-treated (60 μM) wild-type or mutant embryos, shown in dorsal view, anterior to top. (A) Adaxial (arrows) and paraxial *myod* and *myf5* mRNAs are lost upon SU5402 treatment from 30% to 80 or 90% epiboly (dashes indicate approximate position of the germ ring) but are unaffected by cyA treatment. The anterior mesoderm marker *aplnrb* is normally downregulated in paraxial presomitic cells expressing *myf5* (white dashes) and upregulated in adaxial cells (arrows). Both changes were absent after SU5402 treatment. Schematics illustrate the location of equivalent cell types at two successive stages. CNH, chordoneural hinge (hatched); mpcs, muscle precursor cells; PSM, presomitic mesoderm (brackets). (B) *Smo^b641^* mutants retain pre-adaxial *myod* mRNA at 6ss even after cyA treatment, but lack pre-adaxial *myod* mRNA at 15ss. *ptc1* (*ptch2*) mRNA downregulation shows that both *smo* mutation and cyA treatment (shown after longer colour reaction) fully block Hh signalling throughout the axis. Scale bars: 50 µm.

In vertebrates, a key essential step in skeletal myogenesis is activation of myogenic regulatory factors (MRFs) encoded by the *myf5* and *myod* genes (Hinits et al., 2009, 2011; Rudnicki et al., 1993). In mice, distinct myogenic cell populations initiate *myf5* and *myod* expression in different ways, the genes being induced by distinct signals through different *cis*-regulatory elements in different skeletal muscle precursor cells. Once expressed, these MRF proteins have two functions: to remodel chromatin and directly enhance transcription of muscle genes (reviewed by Buckingham and Rigby, 2014). In zebrafish, as the anteroposterior axis forms and extends, *de novo* induction of *myf5* and *myod* mRNAs in slow and fast muscle precursors occurs in tissue destined to generate each successive somite (Coutelle et al., 2001). Zebrafish myogenesis begins at about 75% epiboly stage when adaxial cells that flank the shield/organiser/nascent notochord (hereafter called pre-adaxial cells; see schematic in Fig. 1A), begin MRF expression (Hinits et al., 2009; Melby et al., 1996). Pre-adaxial cells express both *myf5* and *myod*, converge to form two rows of adaxial cells flanking the notochord, become incorporated into somites and differentiate into slow muscle fibres (Coutelle et al., 2001; Devoto et al., 1996; Weinberg et al., 1996). Loss of either MRF alone is not sufficient to prevent slow myogenesis, but loss of both completely inhibits adaxial slow muscle formation (Hinits et al., 2009, 2011). Dorsal tissue immediately lateral to the pre-adaxial cells, the paraxial mesoderm (Fig. 1A), expresses *myf5* and subsequently generates fast muscle once somites have formed, upregulating *myod* in the process. A key to understanding myogenesis in both paraxial and adaxial cells is thus the mechanism(s) by which *myf5* and *myod* expression is regulated.

Intrinsic factors such as Tbx proteins likely interact with extrinsic positional cues within the embryo to pattern myogenesis. Fgf and Tbx function have long been known to interact to drive early mesendoderm patterning, but how directly they control early embryonic myogenesis remains unclear (Kimelman and Kirschner, 1987; Showell et al., 2004; Slack et al., 1987). Various Fgf family members are expressed close to myogenic zones during vertebrate gastrulation (Isaacs et al., 2007; Itoh and Konishi, 2007; Wilkinson et al., 1988). In zebrafish, Fgf signalling is required for mesendoderm formation, tailbud outgrowth and normal fast myogenesis (Draper et al., 2003; Griffin et al., 1995; Groves et al., 2005; Reifers et al., 1998; Yin et al., 2018). Fgf signalling is also involved in early expression of *myf5* and *myod* in pre-adaxial cells, but the mechanism of initial induction of *myf5* and *myod* is unknown (Ochi et al., 2008). Expression of several Fgfs has been detected in the chordoneural hinge (CNH; Fig. 1A) adjacent to pre-adaxial cells (Draper et al., 2003; Groves et al., 2005; Thisse and Thisse, 2005). Subsequently, Hh signalling from the ventral midline maintains MRF expression and progression of the pre-adaxial cells into terminal slow muscle differentiation (Coutelle et al., 2001; Hirsinger et al., 2004; Lewis et al., 1999). Here, we show how both Fgf and Hh extracellular signals cooperate with Tbx genes to control fast and slow myogenesis. In the trunk, Fgf signalling is required for the initiation of myogenesis and acts in cooperation with Tbx16/Tbxta function directly on *myf5* and *myod*. In the tail, by contrast, direct MRF gene induction by Fgf is not required and the evolutionary novelty of midline-derived Hh signalling accounts for adaxial myogenesis.

## RESULTS

### Fgf signalling is essential for induction of adaxial *myf5* and *myod* expression

Adaxial myogenesis is driven by successive Fgf and Hh signals. When Hh signalling was prevented with the Smoothened (Smo) antagonist cyclopamine (cyA) from 30% epiboly, *myf5* and *myod* mRNAs in pre-adaxial cells were unaffected at 90% epiboly (Fig. 1A). In contrast, when Fgf signalling was inhibited with SU5402 from 30% epiboly both *myf5* and *myod* mRNAs were lost (Fig. 1A) (Ochi et al., 2008). Persistence of the anterior mesodermal marker *aplnrb* (Zeng et al., 2007) showed that the lack of MRFs was not due to failure of gastrulation caused by SU5402 treatment (Fig. 1A, Fig. S1; see Table S1 for quantification and Table S5 for a summary checklist of results of this and subsequent experiments). In SU5402-treated embryos, *aplnrb* mRNA revealed the normal invagination of mesoderm and *aplnrb*-expressing cells flanking the organiser. However, both downregulation of *aplnrb* mRNA in paraxial trunk mesoderm that normally expresses *myf5* alone, and pre-adaxial *aplnrb* upregulation were absent in SU5402-treated embryos (Fig. 1A). Thus, early Fgf signalling is required for the initiation of skeletal myogenesis in future trunk regions.

As trunk myogenesis proceeds, Hh signalling becomes essential for adaxial slow myogenesis. Blockade of Hh signalling with cyA or in *smo* mutants (which lack Smoothened, an essential component of the Hh signal transduction pathway), led to loss of *myod* mRNA from adaxial slow muscle at the 6 somite stage (ss), whereas paraxial fast muscle precursors were unaffected (Fig. 1B). Nevertheless, *myod* mRNA transiently accumulated in pre-adaxial cells of presomitic mesoderm (PSM) destined to make trunk somites, but was then lost in anterior PSM (Fig. 1B) (Barresi et al., 2000; Lewis et al., 1999; Osborn et al., 2011; van Eeden et al., 1996, 1998). Thus, as suggested previously (Coutelle et al., 2001; Ochi et al., 2008), in the wild-type situation trunk pre-adaxial *myod* expression is maintained and enhanced by Hh. In contrast, during tail myogenesis at 15ss and thereafter, no pre-adaxial *myod* expression was detected in *smo* mutants or cyA-treated embryos (Fig. 1B, Fig. S2). These data suggest that whereas Hh is necessary for induction of adaxial myogenesis in the tail, Fgf-like signals initiate *myod* expression in trunk pre-adaxial cells.

**Fig. 2.**
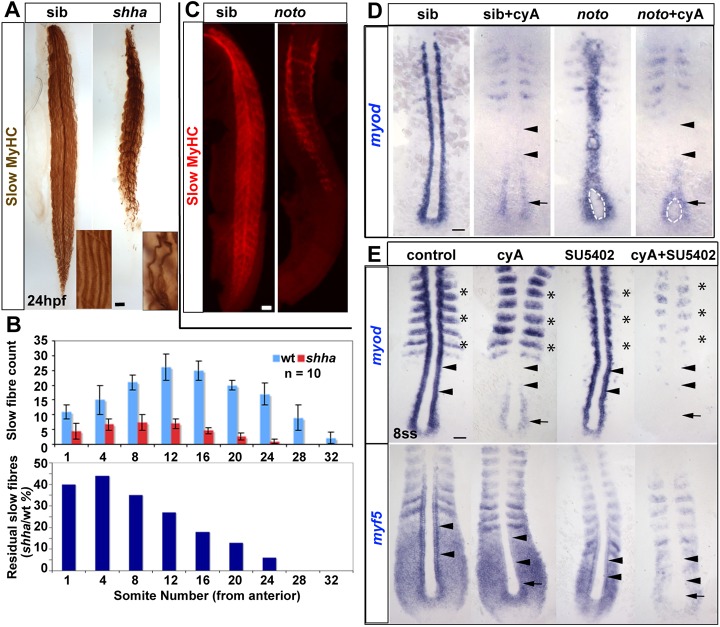
**Successive Fgf and Hh signals drive trunk slow myogenesis.** (A) Immunodetection showing slow fibre reduction is greater in tail than trunk in *shha* mutants. Insets show individual fibres magnified. (B) Quantification of experiment in A. Upper graph shows mean±s.d. (*n*=10 embryos of each genotype). Lower graph shows the fraction remaining in the mutant. (C) Trunk-specific residual slow muscle in *noto* mutant. (D) 5ss embryos from a *noto^n1^* heterozygote incross treated with cyA at 30% epiboly stage, showing loss of adaxial *myod* mRNA in anterior presomitic mesoderm (arrowheads), but retention in the most posterior pre-adaxial mesoderm (arrows) flanking the chordoneural hinge (white outline). (E) Exposure to cyA diminishes *myf5* and *myod* mRNAs in adaxial cells in anterior PSM (arrowheads). Exposure to SU5402 (50 µM) at the tailbud stage ablates residual pre-adaxial *myod* and *myf5* mRNAs in cyA-treated 8ss embryos (arrows). Expression of paraxial *myod* in fast muscle precursors (asterisks) is not affected by cyA but is decreased by SU5402. Scale bars: 50 µm.

Additional evidence emphasises the greater reliance on Hh in tail myogenesis. In *shha* mutants at 24 hours post-fertilisation (hpf), slow muscle was lost from tail but remained in trunk somites, suggesting that slow muscle in tail is more sensitive to reduction in Hh activity (Fig. 2A,B). Moreover, injection of *myod* or *myog* mRNA into embryos lacking Hh signalling was able to rescue slow myogenesis in trunk but not in tail (Fig. S3). Similarly, absence of notochord-derived signals in *noto* (*flh*) mutants, in which the nascent notochord loses notochord character and converts to muscle (Coutelle et al., 2001; Halpern et al., 1995), ablated tail but not trunk slow muscle (Fig. 2C). Treatment of *noto* mutants with cyA shows that *myod* expression is initiated in trunk pre-adaxial cells adjacent to the transient pre-notochordal tissue, but fails to be maintained owing to the blockade of floorplate-derived Hh signals (Fig. 2D). Taken together, these data show that Hh can initiate and then maintain MRF gene expression, but that other signals initiate slow myogenesis in the trunk.

We next tested whether Hh-independent *myod* expression and *myf5* upregulation in trunk pre-adaxial cells requires Fgf signalling. Treatment with cyA left residual pre-adaxial *myod* and *myf5* mRNA flanking the base of the notochord at trunk levels, but ablated adaxial expression in slow muscle precursor cells (Fig. 2E). The residual expression was ablated when, in addition to cyA, SU5402 was used to block Fgf signalling from 30% epiboly (Fig. 2E). Application of SU5402 alone diminished *myf5* and *myod* mRNA accumulation up to the tailbud stage, but caused little if any reduction of adaxial *myf5* and *myod* mRNAs in the tailbud region at 6ss, after midline *shha* function had commenced (Fig. 1B) (Krauss et al., 1993). Nevertheless, SU5402 greatly diminished *myod* expression in somitic fast muscle precursors and reduced the extent of *myf5* expression in paraxial PSM (Fig. 1B) (Groves et al., 2005; Reifers et al., 1998).

### Fgf3, Fgf4, Fgf6a and Fgf8a collaborate to promote MRF expression

To identify candidate Fgf regulators of pre-adaxial myogenesis, the expression patterns of *fgf3*, *fgf4*, *fgf6a* and *fgf8a* were investigated in wild-type embryos (Fig. S4A). As reported previously, *fgf4*, *fgf6a* and *fgf8a* mRNAs were all detected in the posterior dorsal midline at 80% epiboly, followed by *fgf3* in the chordoneural hinge (CNH) and posterior notochord (Fig. S4A) (Kudoh et al., 2001; Thisse and Thisse, 2005; Yamauchi et al., 2009). These Fgfs are candidate regulators of *myf5* and *myod*.

**Fig. 3.**
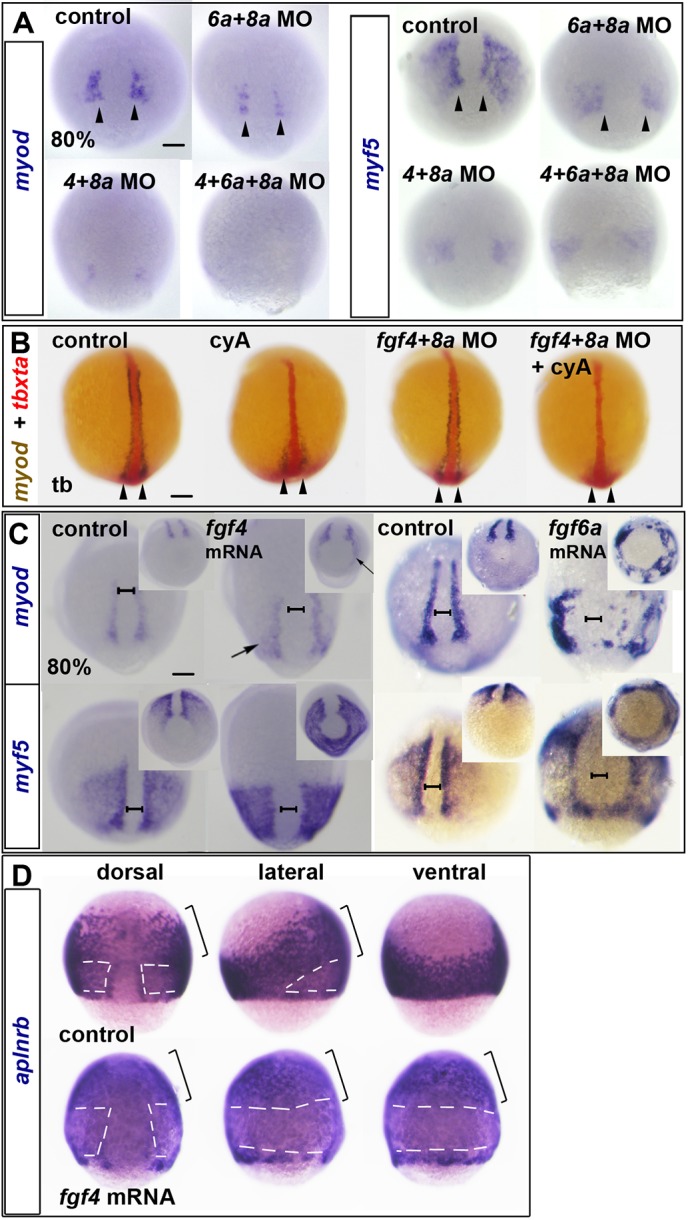
**Dorsally expressed Fgfs drive paraxial myogenesis.** (A-D) ISH for *myod* and *myf5* (A,C) or *aplnrb* (D) mRNAs at 80% epiboly or *tbxta* (red) and *myod* (blue/brown) at the tailbud stage (tb) (B). (A) Reduction of *myod* and *myf5* mRNAs in dual and triple Fgf MO-injected wild-type embryos. Arrowheads indicate nascent adaxial cells. (B) In contrast to 80% epiboly (compare with Fig. S4C), at the tailbud stage, cyA treatment ablates anterior adaxial *myod* mRNA, but leaves pre-adaxial expression intact (arrowheads). Injection of *fgf4*+*fgf8a* MOs ablate residual *myod* mRNA. (C) *fgf4* or *fgf6a* mRNA injection upregulates *myod* and *myf5* mRNAs around the marginal zone (arrows). Note the widening of the unlabelled dorsal midline region (brackets). Insets show the same embryos viewed from the vegetal pole. (D) *fgf4* mRNA injection downregulates *aplnrb* mRNA around the marginal zone (white dashes) but not anteriorly (brackets) in the dorsalised embryo. Scale bars: 100 µm.

Each Fgf was knocked down with previously validated antisense morpholino oligonucleotides (MOs) in wild-type embryos (Fig. S4B). At 80% epiboly, there was little or no decrease of *myf5* or *myod* mRNA in individual Fgf morphants or *fgf8a* mutant embryos (Fig. S4B). Combinatorial knockdown of several Fgfs led to progressively more severe loss of *myod* mRNA and reduction of the raised pre-adaxial and paraxial levels of *myf5* mRNA (Fig. 3A, Fig. S4C,D) and pre-adaxial *aplnrb* mRNA (Fig. S4E). Thus, specific Fgfs collaborate to drive the initial expression of *myod* and *myf5* in pre-adaxial and paraxial cells.

By tailbud stage, however, *fgf4*+*fgf8a* MO treatment alone had little effect on *myod* mRNA accumulation, presumably due to the presence of Hh in the midline (Fig. 3B). Congruently, cyA-treatment reduced anterior adaxial *myod* mRNA, but pre-adaxial expression persisted after blockade of Hh signalling (Fig. 3B). Pre-adaxial *myod* mRNA was ablated in cyA-treated embryos injected with *fgf4+fgf8a* MO (Fig. 3B). Thus, expression of Fgf4 and Fgf8a in the shield, CNH and posterior notochord, provides a spatiotemporal cue for pre-adaxial myogenic initiation in the tailbud.

To test the ability of Fgfs to promote myogenesis further, we generated ectopic Fgf signals by injection of *fgf4* or *fgf6a* mRNA into wild-type embryos, and analysed *myf5* and *myod* mRNA at 80% epiboly (Fig. 3C). Both *myod* and *myf5* mRNAs were upregulated in more ventral regions at levels comparable to those in adaxial cells of control embryos, despite the absence of Hh signalling in these regions. Overexpression of *fgf4* mRNA upregulated *myf5* mRNA in an initially uniform band around the embryo that extended towards the animal pole for a distance similar to that of *myod* mRNA in adaxial cells of controls (Fig. 3C). *myod* was less easily induced, but was upregulated in similar regions of the mesoderm. Fgf4-injected embryos became ovoid, with germ ring constriction that stretched and broadened the notochord (although the numbers of DAPI-stained notochord cells appeared normal). There was a positive correlation between the extent of *myf5* and *myod* mRNA upregulation and the extent of deformation. *aplnrb* mRNA persisted in animal regions of the mesoderm, but was downregulated where *myf5* mRNA was induced nearer to the margin, revealing that anterior/cranial mesoderm is present but resistant to Fgf-driven MRF induction and *aplnrb* suppression (Fig. 3D). Thus, Fgf4 dorsalised the embryo, converting the entire posterior paraxial and ventral mesoderm to a myogenic profile with some regions expressing only *myf5* and others expressing also *myod*, particularly around the germ ring. Fgf6a overexpression also induced ectopic MRFs in cells around the germ ring, which then appeared to cluster (Fig. 3C). Taken together, these data show that posterior/dorsal Fgf signals initiate MRF expression in both pre-adaxial slow and paraxial fast muscle precursors in pre-somitic mesoderm.

### MRFs are initially induced by Fgfs, Tbxta and Tbx16

**Fig. 4.**
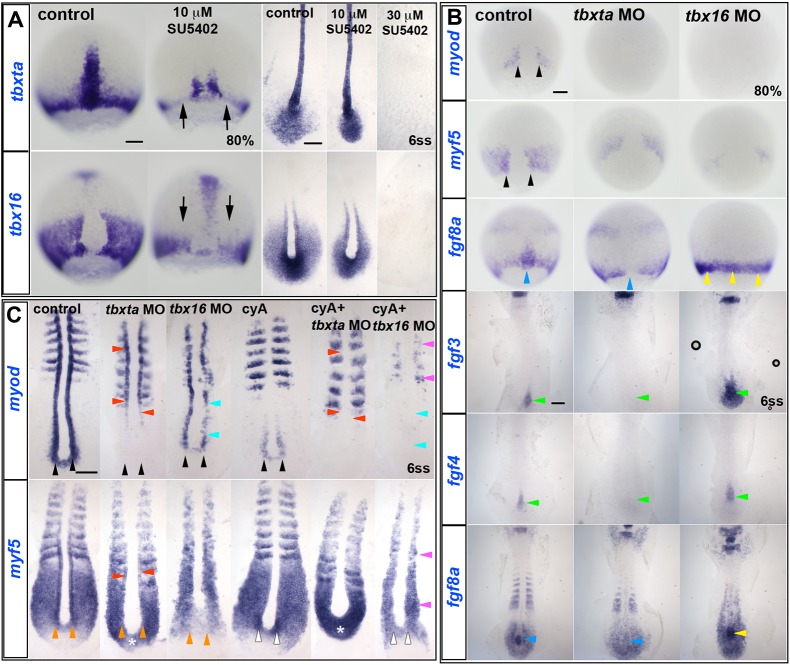
**Redundant Fgf/Tbx and Hh signals are required for MRF induction.** ISH of drug and/or MO-treated embryos. (A) In 10 µM SU5402-treated wild-type embryos, *tbxta* and *tbx16* transcripts are decreased (arrows) at 80% epiboly, but almost normal at 6ss. Both transcripts are absent in 30 µM SU5402-treated embryos at 6ss. (B) Adaxial *myod* expression (black arrowheads) is completely ablated in *tbxta* or *tbx16* morphants at 80% epiboly, and *myf5* expression is greatly decreased (black arrowheads). *fgf8a* mRNA is ablated in the posterior notochord of *tbxta* morphants (blue arrowheads), but upregulated in *tbx16* morphants around the germ marginal zone at 80% and in the posterior notochord at 6ss (yellow arrowheads). Expression of *fgf3* and *fgf4* is absent in posterior notochord of *tbxta* morphants, but enhanced in that location in *tbx16* morphants (green arrowheads). (C) At 6ss, pre-adaxial *myod* expression (black arrowheads) is lost in *tbxta* morphant tailbud, and diminished in *tbx16* morphants. Injection of *tbx16* MO, but not *tbxta* MO, reduces adaxial *myf5* mRNA to the level observed in paraxial mesoderm (orange arrowheads), whereas *tbxta* MO but not *tbx16* MO upregulates *myf5* mRNA in the posterior tailbud (asterisks). *Tbx16* MO abolishes pre-adaxial *myf5* mRNA in cyA-treated embryos (white arrowheads). Adaxial *myf5* and *myod* transcripts recover in *tbxta* morphants, but are ablated by cyA-treatment (red arrowheads). CyA-treatment of *tbx16* morphants ablates adaxial *myod* expression throughout the axis (cyan arrowheads), leaving only residual paraxial *myod* and *myf5* expression (pink arrowheads). Scale bars: 100 µm.

Zebrafish Tbx genes, including *tbxta* and *tbx16* [formerly called *no tail* (*ntla*) and *spadetail* (*spt*), respectively], potentially mediate Fgf signalling in gastrulating embryos (Amaya et al., 1993; Griffin et al., 1995; Smith et al., 1991; Sun et al., 1999). *tbx16* is suppressed by a dominant negative Fgf receptor (FgfR) (Griffin et al., 1998). However, whether *tbxta* and *tbx16* activities are altered by SU5402 treatment, which also blocks FgfR function, is unclear (Rentzsch et al., 2004; Rhinn et al., 2005). Wild-type embryos at 30% epiboly were therefore exposed to SU5402 and subsequently fixed at 80% epiboly or 6ss to investigate expression of *tbxta* and *tbx16* (Fig. 4A). Application of 10 μM SU5402 diminished *tbxta* mRNA in notochord, and *tbxta* and *tbx16* mRNAs in the germ ring (particularly in dorsal paraxial regions) at 80% and in the tailbud at 6ss (Fig. 4A), and 30 μM SU5402 abolished *tbxta* and *tbx16* mRNAs throughout the trunk (Fig. 4A). Thus, Fgf-like signalling is required for normal Tbx gene expression in mesoderm.

Tbxta is required for normal *myf5* and *myod* expression in posterior regions during tailbud outgrowth, partly due to loss of notochordal Hh signalling, which normally maintains a high level of *tbx16* mRNA accumulation in adaxial cells in anterior PSM (Fig. S5) (Coutelle et al., 2001; Weinberg et al., 1996). To test whether Tbx genes are required for MRF expression at earlier stages, each Tbx gene was knocked down and MRF and Fgf expression analysed at 80% epiboly. Tbxta knockdown reduced expression of dorsal midline Fgfs, ablated *myod* mRNA and reduced *myf5* mRNA accumulation in pre-adaxial cells to the level in paraxial regions (Fig. 4B). However, Tbxta knockdown had little effect on either *fgf8a* or *myf5* mRNAs in more lateral paraxial mesoderm or the germ ring (Fig. 4B). This correlation raised the possibility (addressed below) that Tbxta may drive MRF expression through induction of Fgf expression. Tbx16 knockdown, on the other hand, ablated pre-adaxial *myod* mRNA and reduced both pre-adaxial and paraxial *myf5* mRNA without reduction of Fgf expression (Fig. 4B). Indeed, both germ ring *fgf8a* mRNA at 80% epiboly and dorsal midline *fgf3*, *fgf4* and *fgf8a* mRNAs in the tailbud at 6ss appeared to be increased (Fig. 4B), as previously described (Warga et al., 2013). As Tbx16 expression persists in *tbxta* mutants (Amack et al., 2007; Griffin et al., 1998), these data raise the possibility that Tbx16 is required to mediate the action of Fgf signals on myogenesis.

*tbx16* null mutants show a failure of convergent migration of mesodermal cells into the paraxial region (Ho and Kane, 1990; Molven et al., 1990), which, by reducing mesodermal cells flanking the CNH, may contribute to the reduction in MRF mRNAs observed at 80% epiboly. Nevertheless, *tbx16* mutants generate enough paraxial mesoderm that reduced numbers of both paraxial fast muscle and adaxially derived slow muscle fibres arise after Hh signalling commences (Honjo and Eisen, 2005; Weinberg et al., 1996). To investigate whether Tbx16 is required for initial induction of *myf5* and/or *myod* expression in pre-adaxial cells, we titrated MO to reduce *tbx16* function to a level that did not prevent accumulation of significant numbers of trunk mesodermal cells and examined *myf5* and *myod* expression at 6ss (Fig. 4C). In *tbx16* morphants, *myod* mRNA was readily detected in adaxial cells adjacent to notochordal Hh expression (Fig. 4C, cyan arrowheads). Treatment of these *tbx16* morphants with cyA to block Hh signalling, however, ablated adaxial *myod* expression, leaving only weak *myod* in paraxial somitic fast muscle precursors (Fig. 4C, cyan and pink arrowheads). In contrast, treatment of control embryos with cyA left pre-adaxial *myod* mRNA intact (Fig. 4C, black arrowheads). These findings show that Fgf-driven pre-adaxial *myod* expression flanking the CNH requires Tbx16 function.

Adaxial *myf5* expression also requires Tbx16. Tbx16 knockdown reduced *myf5* mRNA accumulation in the posterior tailbud, and also diminished the upregulation of *myf5* mRNA in pre-adaxial and adaxial cells (Fig. 4C, orange arrowheads). Addition of cyA to *tbx16* morphants had little further effect on *myf5* expression (Fig. 4C, white arrowheads). In contrast, cyA treatment alone reduced adaxial *myf5* mRNA in the anterior PSM, but did not affect the *myf5* upregulation in pre-adaxial cells or tailbud *myf5* expression (Fig. 4C, white arrowheads). Additional knockdown of Tbx16 in cyA-treated embryos prevented pre-adaxial *myf5* upregulation (Fig. 4C, white arrowheads). Thus, Tbx16 is required for Fgf to upregulate both *myf5* and *myod* in pre-adaxial cells.

Both pre-adaxial and anterior PSM adaxial *myod* expression were also absent in *tbxta* morphants, but recovered in somites, again due to midline-derived Hh signalling (Fig. 4C) (Coutelle et al., 2001). In marked contrast, Tbxta knockdown upregulated *myf5* mRNA in the tailbud (Fig. 4C, asterisks), presumably reflecting loss of tailbud stem cells that lack *myf5* mRNA. Taken together, the data strongly suggest that *tbx16* is required for midline-derived Fgf signals to induce *myod* and upregulate *myf5* in pre-adaxial cells in the tailbud. In contrast, the loss of MRF expression in *tbxta* mutants could be simply explained by loss of midline-derived Fgf signals, and/or might require some other Tbxta-dependent process.

### *myf5* and *myod* induction by Fgf signalling requires Tbx16

**Fig. 5.**
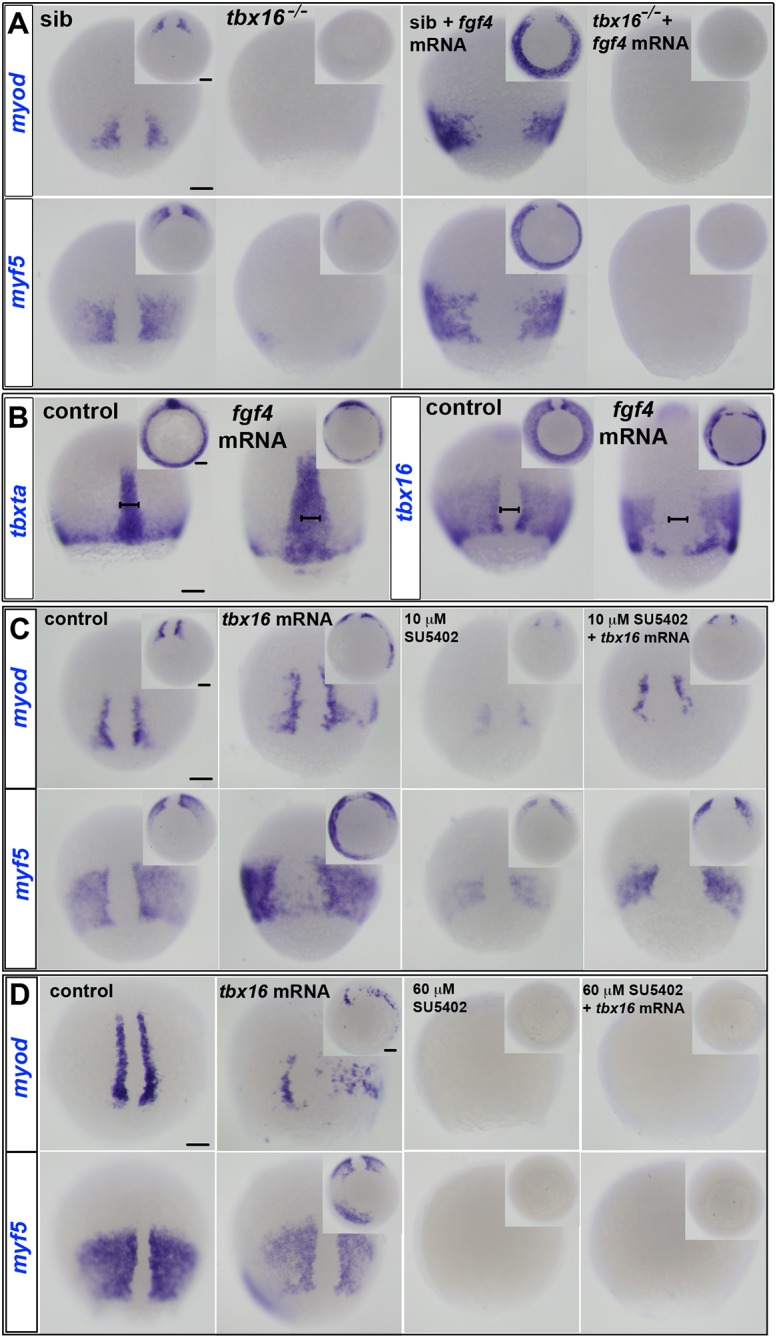
**Tbx16 is necessary and sufficient for MRF induction.** ISH of manipulated embryos at 80% epiboly stage. Dorsal views. Insets show ventral views. (A) *myf5* and *myod* mRNAs flank the dorsal midline in siblings (sib), but are absent or greatly diminished in *tbx16^−/−^* mutants. *fgf4* mRNA injection widened notochord and induced ectopic *myod* and *myf5* mRNA around the germ marginal zone of siblings, but did not rescue expression in *tbx16^−/−^* embryos. (B) *tbxta* mRNA reveals widened notochord (bars) in wild-type embryos injected with *fgf4* mRNA. Both *tbxta* and *tbx16* mRNAs show clumping in the germ ring after overexpression of *fgf4*. (C,D) ISH at 80% epiboly stage for *myf5* and *myod* mRNAs in wild-type control or *tbx16* mRNA-injected embryos treated with SU5402 at 10 µM (C) or 60 µM (D). *myf5* and *myod* mRNAs are ectopically induced in posterior mesoderm by Tbx16 expression, but decreased by administration of 10 µM SU5402 in wild type, and rescued in SU5402-treated embryos by overexpression of *tbx16*. High dose SU5402 prevents MRF expression, even after *tbx16* mRNA injection. Scale bars: 100 µm.

To test rigorously whether Tbx16 is required for Fgf to induce MRFs, *fgf4* mRNA was injected into embryos from a *tbx16* heterozygote incross. Whereas Fgf4 upregulated *myf5* and *myod* mRNAs all around the germ ring in siblings, in sequence-genotyped *tbx16* mutant embryos no upregulation was detected (Fig. 5A). It is clear that mesoderm was present in *tbx16* mutants because the mRNAs encoding Aplnrb, Tbxta, Tbx16 and Tbx16-like (formerly known as Tbx6 and then Tbx6-like) are present in *tbx16* mutants (Fig. S6; Griffin et al., 1998; Morrow et al., 2017). The effect of Fgf4 does not appear to act by radically altering *tbxta* or *tbx16* gene expression (Fig. 5B). Thus, Tbx16 is required for Fgf-driven expression of MRFs in pre-somitic mesoderm.

### Tbx16 requires Fgf-like signalling to rescue *myf5* and *myod* expression

The results so far show that *tbx16* function is necessary for Fgf to induce *myf5* and *myod* (Fig. 5A). To determine whether increased Tbx16 activity is sufficient to induce MRFs, Tbx16 was overexpressed. Injection of *tbx16* mRNA caused ectopic expression of both *myf5* and *myod* in the germ marginal zone (Fig. 5C). Notably, Tbx16 overexpression induced *myf5* mRNA in a much broader region than was observed for *myod* mRNA.

We next tested whether Tbx16 could induce expression of *myf5* or *myod* in the absence of Fgf signalling. Exposure to a high dose of SU5402, which downregulates endogenous *tbx16* and *tbxta* mRNA (Fig. 4A), prevented MRF induction by injection of *tbx16* mRNA (Fig. 5D). Nevertheless, when *tbx16* mRNA was injected into low dose (10 µM) SU5402-treated embryos, which normally have reduced MRF expression, the level of *myf5* and *myod* mRNAs was rescued (Fig. 5C). However, *tbx16* mRNA was less effective at ectopic MRF induction in the presence of SU5402 (Fig. 5C). These results demonstrate that Fgf signalling cooperates with Tbx16 activity in inducing expression of *myf5* and *myod* in pre-adaxial cells at gastrulation stages. Moreover, Tbx16 requires Fgf-like signals to induce MRF gene expression.

### *myf5* and *myod* are direct transcriptional targets of Tbx16

**Fig. 6.**
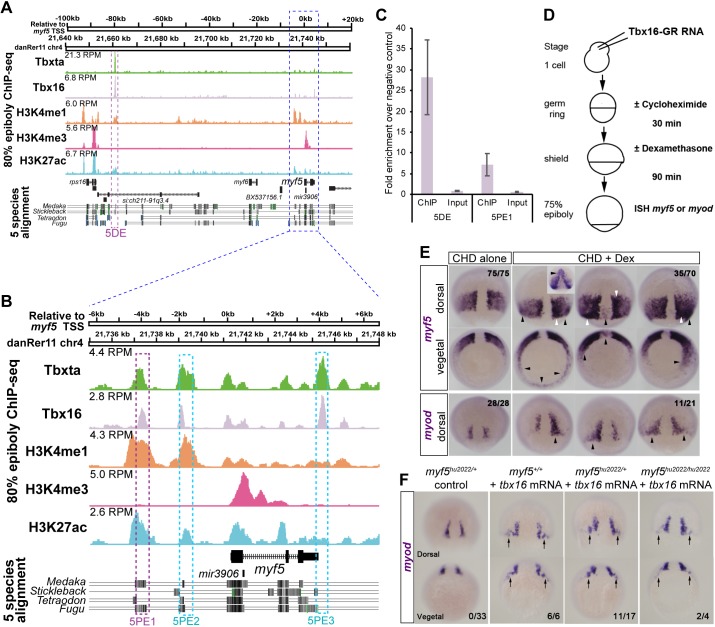
***myf5* is a direct transcriptional target of Tbx16.** (A,B) ChIP-seq on wild-type embryos at 75-85% epiboly reveals endogenous Tbx16 and Tbxta binding events within 120 kb flanking the *myf5* transcriptional start site (TSS). H3K4me3 indicates TSSs. H3K4me1 indicates putative enhancers. H3K27ac indicates active enhancers. Known transcripts with exons (black) and introns (arrowheads) are indicated. Purple and cyan boxes indicate validated and other mentioned Tbx16 binding sites, respectively. (C) ChIP-qPCR validation of Tbx16 peaks on *myf5* distal element (5DE) and proximal element 2 (5PE1). Error bars indicate s.e.m. for biological triplicate experiments. (D) Schematic of Tbx16 direct-target assay. (E) Wild-type embryos injected with *tbx16-GR* mRNA treated with CHD±DEX. CHD alone permits wild-type *myf5* expression at 75-80% epiboly, whereas CHD+DEX induced mosaic ectopic *myf5* expression with strong (white arrowheads, comparable to wild-type pre-adaxial level) and weak (black arrowheads, comparable to wild-type paraxial level) staining. Numbers indicate the fraction of embryos with the expression pattern(s) shown. Inset shows an unusual induction of *myf5* in anterior regions that was not observed with *myod*. (F) Injection of *tbx16* mRNA (200 pg) into embryos from a *myf5^hu2022/+^* heterozygote incross led to ectopic upregulation of *myod* mRNA in the dorsal germ ring (arrows) irrespective of genotype. Numbers indicate fraction of embryos showing ectopic mRNA/total analysed. Scale bars: 100 µm.

In order to determine further the regulatory relationship between Tbx16 and *myf5* and *myod*, we interrogated our previously published chromatin immunoprecipitation and sequencing (ChIP-seq) experiments for endogenous Tbx16 at 75-85% epiboly stage (see Materials and Methods) (Nelson et al., 2017). Our analyses revealed a highly significant peak at −80 kb upstream (*myf5* distal element, 5DE) and two peaks proximal to *myf5* (proximal elements, 5PE1,5PE3) (Fig. 6A,B; Table S3). Cross-referencing to published histone modification ChIP-seq data (Bogdanovic et al., 2012) revealed that 5DE and 5PE1 overlapped significant H3K27ac and H3K4me1 peaks (Table S3), suggesting that these are functionally active enhancers. Tbx16 ChIP-qPCR confirmed the validity of the 5DE and 5PE1 ChIP-seq peaks (Fig. 6C). These putative enhancers are likely to regulate *myf5*, the promoter of which has a H3K4me3 mark, rather than the adjacent *myf6* gene, which is not expressed at 80% epiboly and does not have a H3K4me3 mark. Each peak showed significant conservation to regions adjacent to *myf5* in other fish species (Fig. 6A,B). Thus, ChIP-seq peaks corresponding to histone marks indicative of enhancer activity suggest evolutionarily conserved mechanisms of *myf5* regulation in fish.

We next tested whether Tbx16 is able to positively regulate *myf5* directly by using a dexamethasone (DEX)-inducible system to activate Tbx16 in the absence of translation (Kolm and Sive, 1995; Martin and Kimelman, 2008) (Fig. 6D). Ectopic expression of *myf5* around the germ ring, and of *myod* in a narrower domain flanking the base of the notochord, was observed upon Tbx16 activation (Fig. 6E). Interestingly, across the set of cycloheximide (CHD)+DEX-treated embryos, ectopic *myf5* mRNA was induced to a higher level than elsewhere (as assessed by staining intensity) in a similar region to ectopic *myod* mRNA, suggesting that Tbx16 was able to induce two aspects of pre-adaxial character (*myod* expression and upregulation of *myf5*) directly in this region of the embryo. To confirm this result, we tested whether Myf5 is required for Tbx16 to induce *myod* expression. When *tbx16* mRNA was injected into *myf5* mutant or heterozygote embryos, ectopic *myod* mRNA was observed flanking the base of the notochord in about 50% of mutant or heterozygote embryos, but appeared to be more readily induced in wild-type siblings (Fig. 6F). Thus, Tbx16 is necessary for MRF expression and can induce both *myf5* and *myod* independently, as long as Fgf signalling is active. Moreover, a feed-forward mechanism operates by which Tbx16 induction of *myf5* augments, but is not essential for, induction of *myod*. In summary, Tbx16 directly induces MRF expression in gastrulating mesoderm and is particularly potent in the pre-adaxial region that normally retains high *tbx16* expression.

### Tbxta is essential for pre-adaxial but not paraxial myogenesis

Whereas the entire paraxial PSM expresses *myf5*, pre-adaxial cells upregulate *myf5* and are the first cells to express *myod*. Tbxta and Tbx16 have similar DNA-binding recognition sequences (Garnett et al., 2009; Nelson et al., 2017). Congruently, we observed a prominent Tbxta ChIP-seq peak at the 5DE −80 kb site upstream of *myf5*, and minor peaks at the proximal sites (Fig. 6A,B; Table S3). Because of the role of Tbx16 and Tbxta in *myod* expression, we also examined the *myod* locus for Tbx protein binding. We found multiple sites occupied by Tbxta and Tbx16 either individually or in combination (Table S3). Notably, only one site (DDE3) displayed strongly significant H3K4me1 and H3K27ac peaks and this was only occupied by Tbxta and not by Tbx16 (Fig. S7, Table S3). However, an additional site (DDE1) showed significant occupancy by Tbx16 and Tbxta concurrent with H3K4me1 but not H3K27ac (Fig. S7, Table S3). These findings indicate that differential direct binding of Tbxta and Tbx16 may control both *myf5* and *myod* expression at the inception of skeletal myogenesis.

**Fig. 7.**
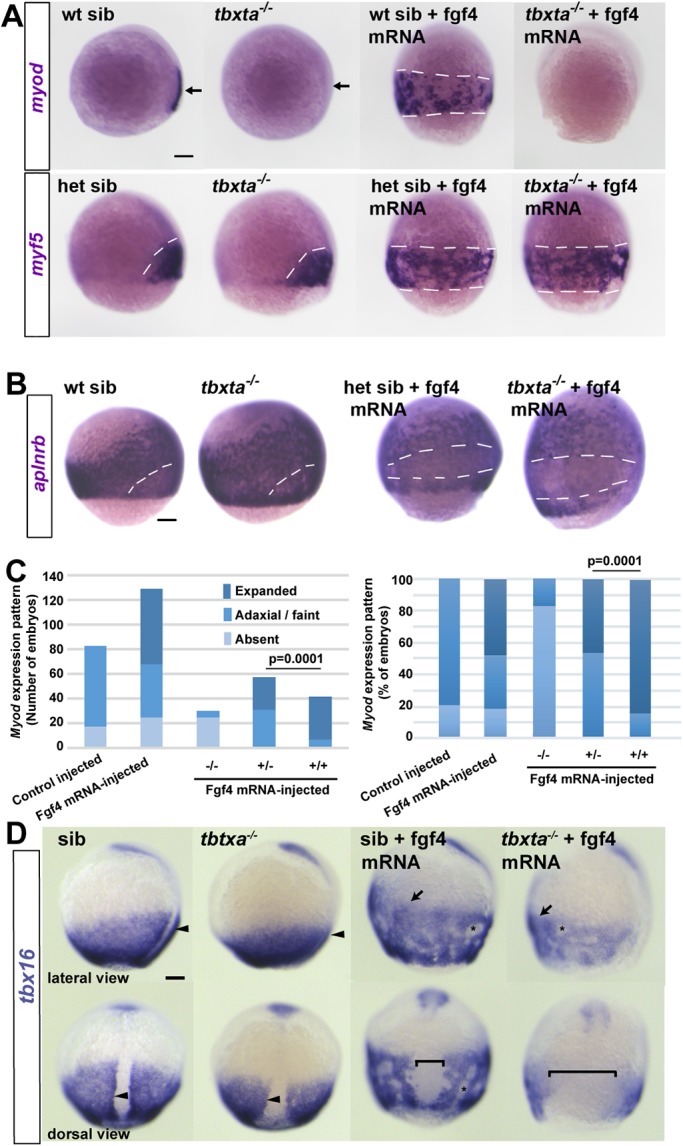
**Tbxta is essential for Fgf4-driven induction of *myod* but not *myf5*.** Embryos from a *tbxta^+/−^* incross injected with 150 pg *fgf4* mRNA or control. (A) *Tbtxa^−/−^* mutants lack *myod* mRNA (arrows) but retain *myf5* mRNA in presomitic mesoderm (white dashes). Fgf4 induced *myf5* and *myod* mRNAs throughout the posterior mesoderm in siblings (sib; white dashes), but failed to induce *myod* mRNA in mutants. (B) Fgf4 suppressed *aplnrb* mRNA in posterior mesoderm above the germ ring (white dashes) in both *tbxta^−/−^* mutants and siblings. In A,B, individually genotyped embryos are shown in lateral view, dorsal to right. (C) Scoring of *myod* mRNA accumulation in response to Fgf4 in a *tbxta^+/−^* incross. Expanded: ventral expansion, generally all around germ ring as in A. Adaxial/faint: either wild-type pattern, or reduced intensity in a small proportion of mutants that was not significantly altered by Fgf4. Left: absolute number of embryos analysed from two experiments to emphasise the lack of induction in mutants (see Table S3). Right: alternative display to highlight the reduced response in heterozygotes compared with wild type (χ^2^ test). (D) Adaxial upregulation of *tbx16* mRNA is lost in *tbxta^−/−^* mutants (arrowheads). Fgf4 upregulates *tbx16* mRNA throughout ventral posterior mesoderm (arrows) and causes mesodermal cell aggregation (asterisks). *tbxta^−/−^* mutants accumulate less *tbx16* mRNA than siblings and have less expression on the dorsal side (brackets). Scale bars: 100 µm.

Is Tbxta also required for MRF expression in response to Fgf? Overexpression of Fgf4 in *tbxta* mutants successfully induced *myf5* mRNA and suppressed *aplnrb* mRNA widely in the posterior mesoderm except in a widened dorsal midline region, showing that the introduced Fgf4 was active (Fig. 7A,B). However, *myod* expression was not rescued in *tbxta* mutants in the dorsal pre-adaxial region of Fgf4-injected embryos, or elsewhere around the germ ring (Fig. 7A). Moreover, even an increased dose of 225 pg *fgf4* mRNA/embryo failed to rescue *myod* mRNA in *tbxta* mutants. Importantly, *tbxta* heterozygotes showed significantly less extensive induction of *myod* mRNA in response to Fgf4 than did their wild-type siblings (*P*=0.0001 χ^2^-test; Fig. 7C; Table S4). Therefore, Tbxta is essential for *myod* induction in pre-adaxial cells independently of its role in promoting expression of midline Fgfs.

Although Fgf4 injection did not radically alter the location of *tbx16* or *tbxta* mRNA (Fig. 5B), we noticed that the higher level of *tbx16* mRNA in adaxial compared with paraxial cells was not obvious in Fgf4-injected wild-type embryos, with high levels present at all dorsoventral locations, presumably because pre-adaxial character was induced widely in posterior mesoderm (Fig. 5B). Nevertheless, as Fgf4 injection into *tbxta* mutants induced *myf5* but not *myod* mRNA (Fig. 7A), it seems that Tbxta is essential for progression from *myf5* to *myod* expression.

**Fig. 8.**
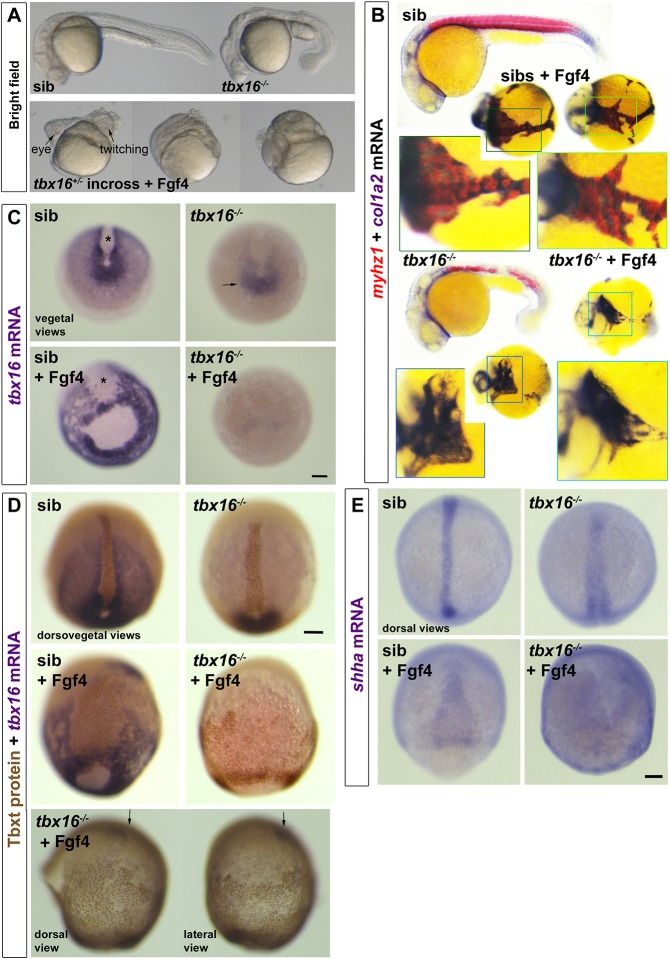
**Tbx16 is essential for Fgf4-driven upregulation of *tbx16* and suppression of *tbxta*.** Embryos from a *tbx16^+/−^* incross injected with 150 pg *fgf4* mRNA or control. (A) By 24 hpf, Fgf4-injected embryos have disorganised heads and, although lacking obvious trunk or tail, some contain twitching muscle. (B) ISH for *col1a2* for dermomyotome/connective tissue and *myhz1* for skeletal muscle revealed muscle in *fgf4*-injected sibs, but not in *tbx16^−/−^* mutants. Boxed areas are magnified to show the alternating pattern of aggregated muscle and connective tissue in Fgf4-injected siblings, but the reduced *col1a2* and absent *myhz1* mRNA in Fgf4-injected mutants. Note the aggregation of posterior mesoderm cells into strands around the yolk. (C) ISH for *tbx16* mRNA in embryos from a *tbx16^+/−^* incross at around 90% epiboly. Nonsense-mediated decay of the mutant transcript is apparent (arrow). *fgf4* RNA injection increases *tbx16* mRNA in paraxial mesoderm, widens dorsal axial notochord domain (asterisks) and causes aggregation of paraxial cells in siblings, but suppresses residual *tbx16* transcript in mutants. (D) Immunodetection of Tbxt protein and *tbx16* mRNA in Fgf4-injected and control embryos from a *tbx16^+/−^* incross. The Fgf4-injected *tbx16*^−/−^ mutant (bottom) reveals nuclear Tbxt protein in the entire posterior mesoderm. Residual *tbx16* mRNA in the prechordal region (arrows) but absence in posterior mesoderm demonstrates the genotype. (E) Widespread upregulation of *shha* mRNA reveals the notochord-like character of posterior mesoderm in Fgf4-injected *tbx16*^−/−^ mutant. Scale bars: 100 µm.

Two hypotheses could explain the lack of *myod* expression in Fgf4-injected *tbxta* mutants. First, despite the apparent lack of Tbxta protein in adaxial cells (Ochi et al., 2008; Schulte-Merker et al., 1994a), Tbxta might act directly on *myod*. Alternatively, Fgf-driven Tbxta activity might act indirectly in pre-adaxial cells to upregulate Tbx16 and thereby drive *myod* expression. We therefore examined the ability of Fgf4 to upregulate Tbx16 in *tbxta* mutants. Fgf4 enhanced *tbx16* mRNA throughout the posterior mesoderm in siblings, with the exception of the widened notochordal tissue that contained nuclear Tbxta protein and failed to upregulate MRFs (Figs 7D and 8D). In *tbxta* mutants, Fgf4 also enhanced *tbx16* mRNA in the ventral mesoderm, but a broader dorsal region did not express *tbx16*. Moreover, the level of *tbx16* mRNA appeared to be lower than in siblings (Fig. 7D). Thus, Fgf4-injected *tbxta* mutants lack both Tbxta and Tbx16 upregulation in pre-adaxial cells. We conclude that, whereas Fgf-driven induction of lateral myogenic tissue requires Tbx16 but not Tbxta, induction of pre-adaxial character (marked by upregulated *myf5* and *myod* mRNAs) requires both Tbx genes.

### Fgf action on Tbx16 suppresses dorsoposterior axial fate

Finally, we examined the wider effect of Fgf signalling when skeletal muscle cannot form in the absence of *tbx16* function. Excess Fgf action in embryos causes gross patterning defects (Kimelman and Kirschner, 1987; Slack et al., 1987). When Fgf4 was overexpressed in *tbx16* heterozygote incross lays, some anterior mesodermal tissue formed and head tissues, such as eye and brain, were apparent, but trunk and tail mesoderm was grossly deficient (Fig. 8A). In siblings overexpressing Fgf4, some muscle was formed and truncated embryos were observed to twitch at 24 hpf. In contrast, no muscle was detected in *tbx16* mutants overexpressing Fgf4 (Fig. 8B). Moreover, the residual expression of mutant *tbx16* mRNA at 90% epiboly observed in uninjected embryos was lost upon Fgf4 overexpression (Fig. 8C). This suggests that the cells with tailbud character normally accumulating in *tbx16* mutants were missing. Instead, widespread expression of Tbxta protein in nuclei far from the germ ring suggested that the entire posterior (but not anterior) mesoderm had converted to notochord, the most dorsal posterior mesoderm fate (Fig. 8D). Indeed, *shha* mRNA, a marker of notochord, was found to be broadly but weakly upregulated around the embryo in *tbx16* mutant embryos injected with *fgf4* mRNA, but not in their siblings (Fig. 8E). The data suggest that Fgf4 drives early involution of all posterior mesoderm precursors, leaving none to form a tailbud. In the presence of Tbx16, Fgf4 also dorsalises most involuted trunk mesoderm to muscle, whereas in the absence of Tbx16 Fgf4 converts most of the trunk mesoderm to notochord precursors.

## DISCUSSION

The current work contains four main findings. First, that Tbx16 directly binds and activates *myf5* regulatory elements to initiate skeletal myogenesis. Second, that Fgf signalling acts through Tbx16 to drive the initial myogenic events in the adaxial cell lineage, which subsequently require Hh signalling to complete myogenesis. Third, that Tbxta, the dorsal-most/posterior Tbx factor, binds directly to *myod* regulatory elements and also promotes dorsal midline expression of Fgfs, which subsequently cooperate to drive dorsal myogenesis through Tbx16. Fourth, that Fgf action through Tbx16 suppresses the dorsoposterior axial fate induced by Tbxta. Overall, Tbx transcription factors provide a crucial link between mesoderm induction and the initiation of myogenesis, which has profound implications for understanding the evolution of vertebrates.

### Tbx genes and myogenesis

Building on previous evidence that Tbx16 upregulates *myf5* expression (Garnett et al., 2009; Mueller et al., 2010), our findings show that *myf5* and *myod* genes are direct targets of Tbx16. We also present evidence that *myf5* and *myod* are direct targets of Tbxta. As MRF gene activity drives commitment to skeletal myogenesis in vertebrates, our findings place Tbx protein activity at the base of skeletal myogenesis in zebrafish (Fig. S8).

Before myogenesis, Tbx16 is required for migration of most trunk PSM cells away from the ‘maturation zone’ immediately after their involution (Griffin and Kimelman, 2002; Row et al., 2011). Our analysis of *aplnrb*-expressing mesodermal cells shows that most anterior (i.e. head) and posterior ventral (i.e. ventral trunk) mesoderm involution and migration occurs normally in both *tbx16* and *tbxta* mutants. Indeed, some PSM eventually yielding muscle is formed in *tbx16* mutant trunk (Amacher et al., 2002; Kimmel et al., 1989). PSM is more severely lacking in *tbx16;tbxta* or *tbx16;tbx16l* double mutants (Amacher et al., 2002; Griffin et al., 1998; Morrow et al., 2017; Nelson et al., 2017) or after Tbxtb knockdown in the *tbxta* mutant (Martin and Kimelman, 2008). Cooperation of Tbx proteins in PSM formation also occurs in *Xenopus* (Gentsch et al., 2013). It is likely, therefore, that all PSM formation and its accompanying *myf5* expression requires Tbx proteins, which may help explain why *tbx16* mutants have increased pronephric mesoderm (Warga et al., 2013). As Tbx16 is required for direct induction of *myf5* and for *pcdh8*, *msgn1*, *mespaa* and *tbx6* expression in PSM (Fior et al., 2012; Goering et al., 2003; Griffin and Kimelman, 2002; Lee et al., 2009; Morrow et al., 2017; Yamamoto et al., 1998), the data support previous proposals (Amacher and Kimmel, 1998; Griffin and Kimelman, 2002) of a role for Tbx16 in promotion of the earliest step in PSM formation, en route to myogenesis. These early actions of Tbx16 and Tbxta proteins have previously masked their direct myogenic actions in mutants.

Our data argue that once posterior (i.e. trunk) mesoderm forms, Tbx proteins are still required for MRF expression and normal myogenesis. Hh signalling from notochord acts to maintain adaxial MRF expression in wild type and, if Tbx-driven initiation fails, Hh can initiate *myod* and upregulate *myf5* expression, thereby driving slow myogenesis (Blagden et al., 1997; Coutelle et al., 2001; Du et al., 1997). When Hh signalling is prevented, both Tbx16 and Tbxta are essential for initial pre-adaxial *myod* transcription. Conversely, Tbx16-induced ectopic *myod* expression is restricted to a narrower mesodermal region flanking the pre-adaxial cells, likely due to the restricted expression of *smarcd3* (Baf60c) in this region (Ochi et al., 2008). Nevertheless, *myod* is induced by Tbx16 in the absence of Myf5, probably through direct binding to regulatory elements in the *myod* locus. It is possible that changes in chromatin structure in *myf5* and *myod* loci accompanying posterior mesoderm formation facilitate Tbx16 access to its binding sites in these MRF genes.

Adaxial slow and paraxial fast myogenesis differ (Blagden et al., 1997; Devoto et al., 1996). Paraxial PSM expresses *myf5* after involution, which requires Tbx16, but not Tbxta. However, fast myogenesis is delayed until somites form, perhaps through Tbx6 action (Windner et al., 2015). In contrast, pre-adaxial cells that generate slow muscle within PSM require both Tbxta and Tbx16 function for upregulation of *myf5* and initiation of *myod* expression. Thus, distinct Tbx proteins are required for normal adaxial and paraxial myogenesis.

Residual muscle in *tbx16* mutants is likely driven by Tbx16l (Morrow et al., 2017). However, as *tbx16;tbx16l* double mutants continue *myod* mRNA expression at a reduced level throughout the axis at 24 hpf (Morrow et al., 2017), we predict this is in adaxially derived slow muscle induced by Hh signalling.

To identify likely MRF enhancers at 80% epiboly, we have largely restricted our focus to robust Tbx16 and Tbxta ChIP-seq peaks that are co-incident with established histone marks indicative of active enhancers, H3K4me1 and H3K27ac. As many transcription factor-binding events may be non-functional, not all enhancers have H3K27ac (Pradeepa, 2017; Pradeepa et al., 2016) and a minority of embryonic cells are myogenic, additional Tbx16 and Tbxta ChIP-seq peaks beyond 5DE, 5PE1 and DDE3 may mark functionally important enhancers regulating *myf5* and *myod*. Of particular note DDE1, −31 kb upstream of *myod*, with Tbx16 and Tbxta ChIP-seq peaks that colocalise with significant H3K4me1, may be functionally important.

Understanding of the elements driving specific aspects of zebrafish *myf5* expression is limited. As with murine *Myf5* (Buckingham and Rigby, 2014), our data suggest that elements ∼80 kb upstream of the transcription start site are required for *myf5* expression. A BAC transgenic encompassing 5DE drives GFP expression in muscle, although analysis of shorter constructs has been confounded by cloning artefacts (Chen et al., 2001, 2007). We also observe Tbx-binding peaks far upstream of zebrafish *myod*. Upstream elements are known to initiate murine *Myod* (*Myod1*) expression in some embryonic regions, but whether these elements drive the earliest myotomal regulation of *Myod* is unknown (Chen and Goldhamer, 2004). Mouse *Tbx6* and Tbxt family genes are also required for trunk/tail, but not cranial, myogenesis (Nandkishore et al., 2018). Moreover, other Tbx proteins bind similar DNA motifs and are required to pattern cranial, cardiac and limb muscle (Don et al., 2016; Knight et al., 2008; Lu et al., 2017; Papaioannou, 2014; Valasek et al., 2011). The extent to which Tbx genes act through similar binding elements to initiate MRF expression and myogenesis across vertebrates remains to be determined.

### Fgf and myogenesis

Tbx16 is required for Fgf signalling to induce *myf5* (Fig. 5A, Fig. S8). In its absence, Fgf drives all posterior mesoderm towards a notochord-like fate (Fig. 8D,E), probably via activation of Tbxta. Fgf signalling is required for expression of *myf5* in tailbud paraxial PSM (Groves et al., 2005). Here, we show that Fgf is also required for the earliest *myf5* expression in involuting trunk mesoderm and for the initiation of *myf5* and *myod* expression in pre-adaxial cells destined to form the slow muscle of anterior somites. Our data provide further evidence that this MRF expression is subsequently maintained by Hh signalling, as the shield/tailbud-derived sources of Fgf recede from the adaxial cells (Coutelle et al., 2001; Osborn et al., 2011) (Fig. S8). We find that Fgf action is stronger in trunk (as opposed to tail) somites, consistent with (1) dwindling Fgf mRNA levels as tailbud outgrowth slows and (2) the unresolved issue of Hh-independent initiation of MRF expression in the anterior-most somites of murine smoothened mutants (Zhang et al., 2001). We suggest this MRF expression is triggered by Fgf in mouse, as in zebrafish.

Our data argue strongly that Fgf signalling not only promotes *tbx16* expression, but also enhances the activity of the Tbx16 protein, constituting a feed-forward mechanism. The MRF-inducing activity of Tbx16 is suppressed by inhibition of Fgf signalling (Fig. 5C,D). Bearing in mind the existence of Tbx16l and Tbxta, this result is consistent with the finding that *tbxta* or *tbx16* mutation sensitises embryos to Fgf inhibition (Griffin and Kimelman, 2003). As Tbx16 overexpression can expand PSM fates and reverse the effect of partial Fgf inhibition, a primary role of Fgf signalling is to cooperate with Tbx16 to drive expression of its target genes, including *myf5*. This understanding provides mechanistic insight into how the effects of Fgf on gastrulation movements and histogenesis are separated, as originally proposed (Amaya et al., 1993). Interestingly, Tbx16 overexpression rescues *myf5* mRNA preferentially on the dorsal side of the embryo, suggesting that BMP and/or other signals continue to suppress PSM fates ventrally, and thus that Tbx16 does not act by simply suppressing the inhibitory effect of such signals.

Tbxta and Fgf appear to act in a positive-feedback loop to both maintain tailbud character and diversify PSM into pre-adaxial and paraxial. Tbxta is required for normal Fgf signalling from the midline to promote pre-adaxial myogenesis. Fgf overexpression drives *myf5* in *tbxta* mutants, but cannot drive *myod*. ChIP shows sites upstream of *myod* preferentially bound by Tbxta. As Tbxta is not readily immunodetectable in these pre-adaxial cells (Hammerschmidt and Nüsslein-Volhard, 1993; Odenthal et al., 1996; Schulte-Merker et al., 1994a), Tbxta may either open the *myod* locus, act indirectly, or remain tightly bound despite low free concentration. In the absence of Tbx16, Fgf drives the entire germ ring towards notochord fate, preventing continued tailbud outgrowth. It seems, therefore, that Fgf/Tbx16 interaction is required to maintain tailbud stem cells and promote PSM formation. In a chordate ancestor, the evolution of interaction between Fgf/Tbx6/16-dependent muscle-forming tissue and Fgf/Tbxta-dependent dorsal organiser might be a key innovation leading to both tailbud stem cells and notochord.

### Evolution of vertebrates

As efficient motility driven by sarcomeric muscle is found throughout triploblasts, it is likely that mesodermal striated muscle existed in the common ancestor of deuterostomes and protostomes. There is consensus that the appearance of neural crest, notochord and a post-anal tail were significant evolutionary steps for chordates (Gee, 2018). Already in cephalochordates at least two kinds of striated muscle had evolved in anterior somites (Devoto et al., 2006; Lacalli, 2002). Our evidence that initiation of both slow and fast myogenesis in the most anterior trunk is driven by Fgf/Tbx signalling indicates that a major function of this early mesodermal inducer was induction of trunk striated myogenesis, which may constitute an ancestral chordate character. Once Hh is expressed in maturing midline tissues, it triggers terminal differentiation of muscle precursors into functional muscle through a positive-feedback loop (Coutelle et al., 2001; Osborn et al., 2011) (Fig. S8). Parallel diversification of neural tube cells, also regulated by Hh (Placzek and Briscoe, 2018), may have generated matching motoneural and muscle fibre populations that enhanced organismal motility. With the evolution of a *tbxta*-dependent tailbud destined to make the post-anal tail and notochord, our data suggest that weakening Fgf signalling continued to induce *myf5* expression and paraxial mesoderm character through *tbx16*, but was insufficient to induce adaxial myogenesis. The presence of Hh and Tbxta plus Tbx16, however, ensure that adaxial slow muscle is initiated in the zebrafish tail.

In the anterior somites of amniotes, as in zebrafish, Hh signalling maintains, rather than initiates, *myf5* expression (Zhang et al., 2001). In more posterior somites of zebrafish, *Xenopus* and amniotes, Hh signalling drives *myf5* initiation (Borycki et al., 1999; Grimaldi et al., 2004). Compared with zebrafish, however, murine *Myf5* induction is further delayed until after somitogenesis, when Gli3 repressive signals in PSM have diminished (McDermott et al., 2005). In mouse, Tbxt (known as brachyury) is required for myogenesis and binds 20 kb downstream of *Myod*, but does not obviously control its expression (Lolas et al., 2014). No clear orthologue of *tbx16* exists in mammals, although it clusters by sequence with *Tbx6* genes. Mammalian Tbx6 suppresses neurogenesis in posterior paraxial mesoderm, suggesting that additional mechanisms have evolved that suppress early pre-somitic *Myf5* expression (Chapman and Papaioannou, 1998). Indeed, possible low level *Myf5* expression in PSM has long been a source of controversy (George-Weinstein et al., 1996; Gerhart et al., 2004). Thus, there has been diversification in how these Tbx genes regulate somitic myogenesis.

The ancestral situation seems clearer. In amphioxus, *Tbx6/16* is expressed in tailbud and PSM (Belgacem et al., 2011). In *Ciona*, knockdown of Tbx6b/c/d leads to reduced *MyoD* expression, loss of muscle and paralysis (Imai et al., 2006). In *Xenopus*, both Tbx6 and VegT are implicated in early myogenesis (Callery et al., 2010; Fukuda et al., 2010; Tazumi et al., 2010), although some mechanisms may differ from those in zebrafish (Maguire et al., 2012). By adding our zebrafish findings, we show that in all major chordate groups Tbx-dependent gene regulation is central to skeletal myogenesis. The conserved involvement, yet divergent detail, of how *Tbx16*, *Tbx6* and *Tbxt* genes regulate somitic myogenic diversity along the body axis are consistent with selective pressures on these duplicated Tbx gene families playing a key role in the diversification of myogenesis in the vertebrate trunk and tail, characters that gave these chordates their predatory advantage.

## MATERIALS AND METHODS

### Zebrafish lines and maintenance

Mutant lines *fgf8a^ti282a^* (Reifers et al., 1998), *noto^n1^* (Talbot et al., 1995), *smo^b641^* (Barresi et al., 2000), *tbxta^b195^* and *tbx16^b104^* (Griffin et al., 1998) are likely nulls and were maintained on King's wild-type background. Staging and husbandry were as described previously (Westerfield, 2000). All experiments were performed under licences awarded under the UK Animal (Scientific Procedures) Act 1986 and subsequent modifications.

### *In situ* mRNA hybridisation and immunohistochemistry

*In situ* mRNA hybridisation (ISH) for *myf5* and *myod* was performed as described previously (Hinits et al., 2009). Additional probes were: *fgf3* (Maroon et al., 2002), *fgf4* (IMAGE: 6790533, https://www.ncbi.nlm.nih.gov/nuccore/BC128817), *fgf6*a (Thisse and Thisse, 2005), *fgf8a* (Reifers et al., 1998), *tbxta* (Schulte-Merker et al., 1994b) and *tbx16* (Griffin et al., 1998; Ruvinsky et al., 1998). Anti-Ntla immunostaining was performed after ISH using rabbit anti-Ntla (Schulte-Merker et al., 1992; 1:2000) and goat anti-rabbit IgG-HRP-conjugated secondary antibody (PI-1000-1, Vector Laboratories).

### Embryo manipulations

Embryos were injected with MOs (GeneTools LLC) as indicated in Table S2 to *fgf3*, *fgf4*, *fgf6a*, *fgf8a*, *tbxta* (Feldman and Stemple, 2001) or *tbx16* (Bisgrove et al., 2005). Controls were vehicle or irrelevant mismatch MO. Cyclopamine (100 µM in embryo medium), SU5402 (at the indicted concentration in embryo medium) and vehicle control were added at 30% epiboly to embryos whose chorions had been punctured with a 30G hypodermic needle. A PCR product of *fgf4* (IMAGE: 6790533) was cloned (primers in Table S2) into the SacI/SalI sites of pβUT3 to make mRNA for overexpression. One-cell-stage embryos were injected with 100-220 pg *fgf4* mRNA (made with messageMachine), 50 pg *fgf6a* mRNA, 150 pg *tbx16* mRNA (Griffin et al., 1998) or 150 pg *tbx16*-GR mRNA (Jahangiri et al., 2012). For hormone-inducible Tbx16 activation, mRNA corresponding to Tbx16 fused to the hormone-binding domain of glucocorticoid receptor (GR) was overexpressed in wild-type embryos. The resulting protein is held in the cytoplasm until DEX from shield stage stimulates GR nuclear translocation. In the presence of the translation inhibitor CHD (added prior to DEX at the germ ring stage), increased nuclear Tbx16 is expected to induce only direct targets of Tbx16 in a mosaic fashion. Embryos were treated with 10 μg/ml final concentration of CHD 2 h prior to collection at 75-80% epiboly. CHD caused ∼5% delay in epiboly, showing that it was active. After 30 min, 20 μM DEX was added for the remaining 1.5 h.

### ChIP-seq and ChIP-qPCR

ChIP-seq data from GSE84619 and GSE32483 were analysed as reported previously (Nelson et al., 2017). Multiz Alignments & Conservation from UCSC Genome Browser (Haeussler et al., 2019) are shown beneath schematics. ChIP-seq peak height was measured in reads per million reads (RPM). ChIP-qPCR experiments were performed as previously reported (Jahangiri et al., 2012) using the primers in Table S2.

## Supplementary Material

Supplementary information

Reviewer comments
