# Supplementary material for: Fgf-driven Tbx protein activities directly induce myf5 and myod to initiate zebrafish myogenesis
Source: Development. 2020 Apr 28;147(8):dev184689. doi: 10.1242/dev.184689 (PMC7197714; doi:10.1242/dev.184689)
Supplement: Supplementary information [file develop-147-184689-s1.pdf]

Fig. S1

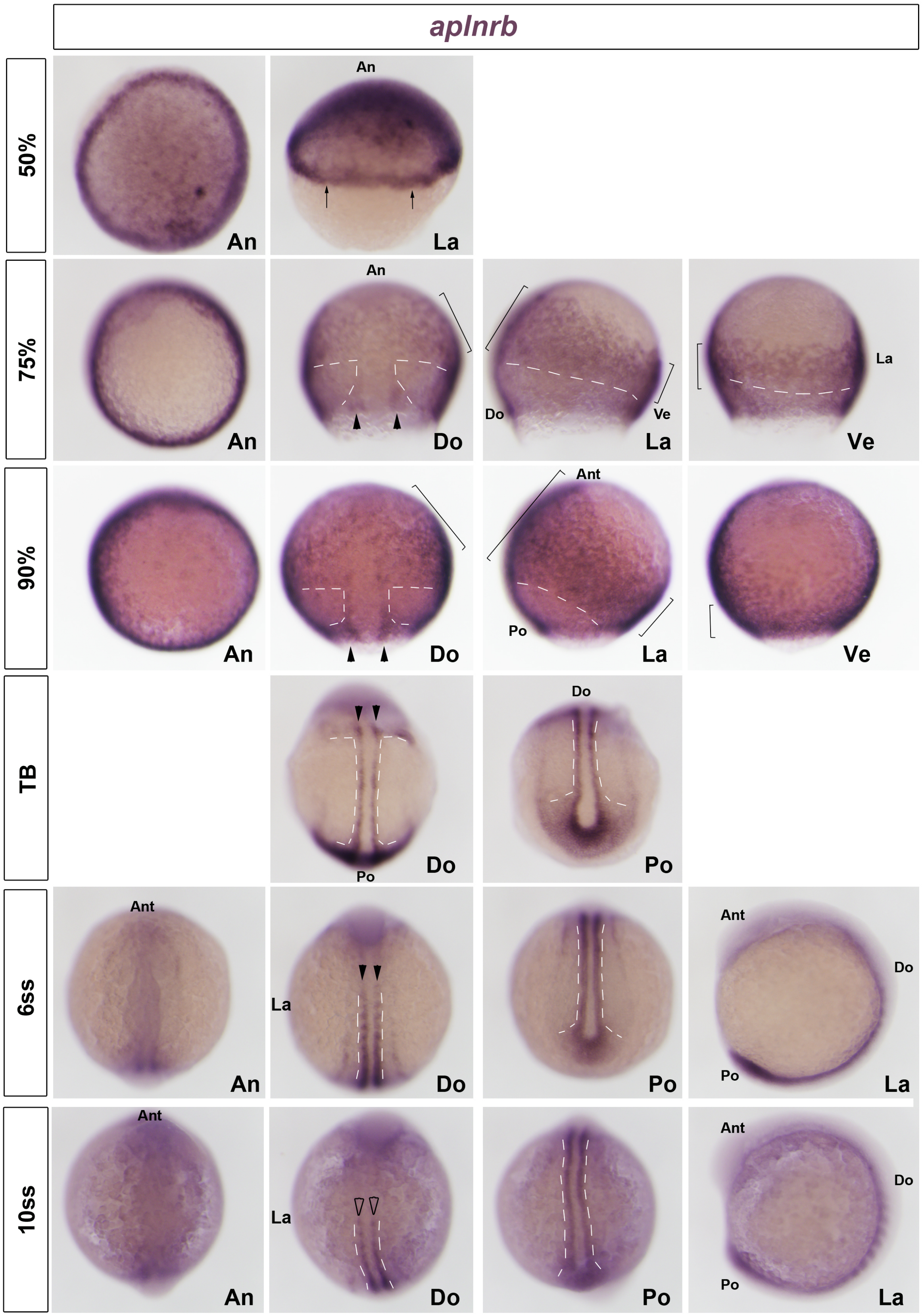

Fig. S1. Expression of *aplnrb* mRNA during zebrafish axis formation.
Wholemount in situ mRNA hybridization of apelin receptor b *(aplnrb)* mRNA in zebrafish embryos at the indicated stages, shown in animal (An), lateral (La), dorsal (Do, animal to top), ventral (Ve, animal to top) and posterior (Po, dorsal to top) views. Ant = anterior. Note significant expression in early germ ring (arrows), future cranial mesoderm (large and small brackets highlight comparable regions of expression) and adaxial cells (arrowheads). Expression is lacking in paraxial mesoderm (white dashes) that expresses *myf5* and later *myod* mRNAs (see Fig. 1C). Between 75-90% epiboly, *aplnrb* mRNA has a complex and informative expression pattern, marking the anterior invaginating mesoderm cells around the germ ring and the pre-adaxial cells, but appears down-regulated in more lateral regions expressing *myf5* but not *myod*.

Development • Supplementary information

Fig. S2

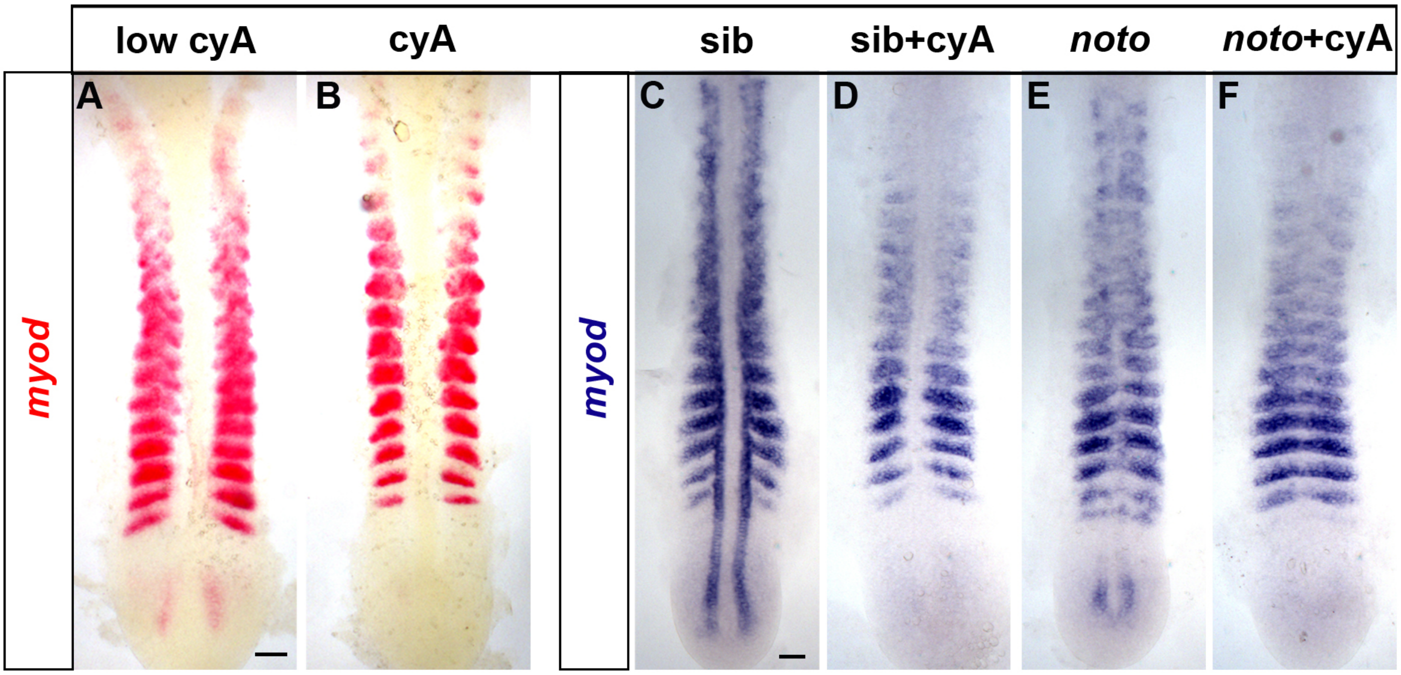

Fig. S2. Hh signals are required for induction of adaxial *myod* in the tail.

Dorsal flatmount preparations showing in situ mRNA hybridisation for *myod* at 15ss. **A,B.** Wild-type embryos were treated with either a low dose (50 µM, A) or the standard dose of cyA (100 µM, B). The control vehicle-treated embryo is shown in Fig. 1B. **C-F.** Hh signalling is required to drive pre-adaxial *myod* induction in the tail of *noto* mutants. Embryos from a $noto^{m614/+}$ incross were treated with cyA (D,F) or vehicle control (C,E). Bars: 50 µm.

Development • Supplementary information

Fig. S3

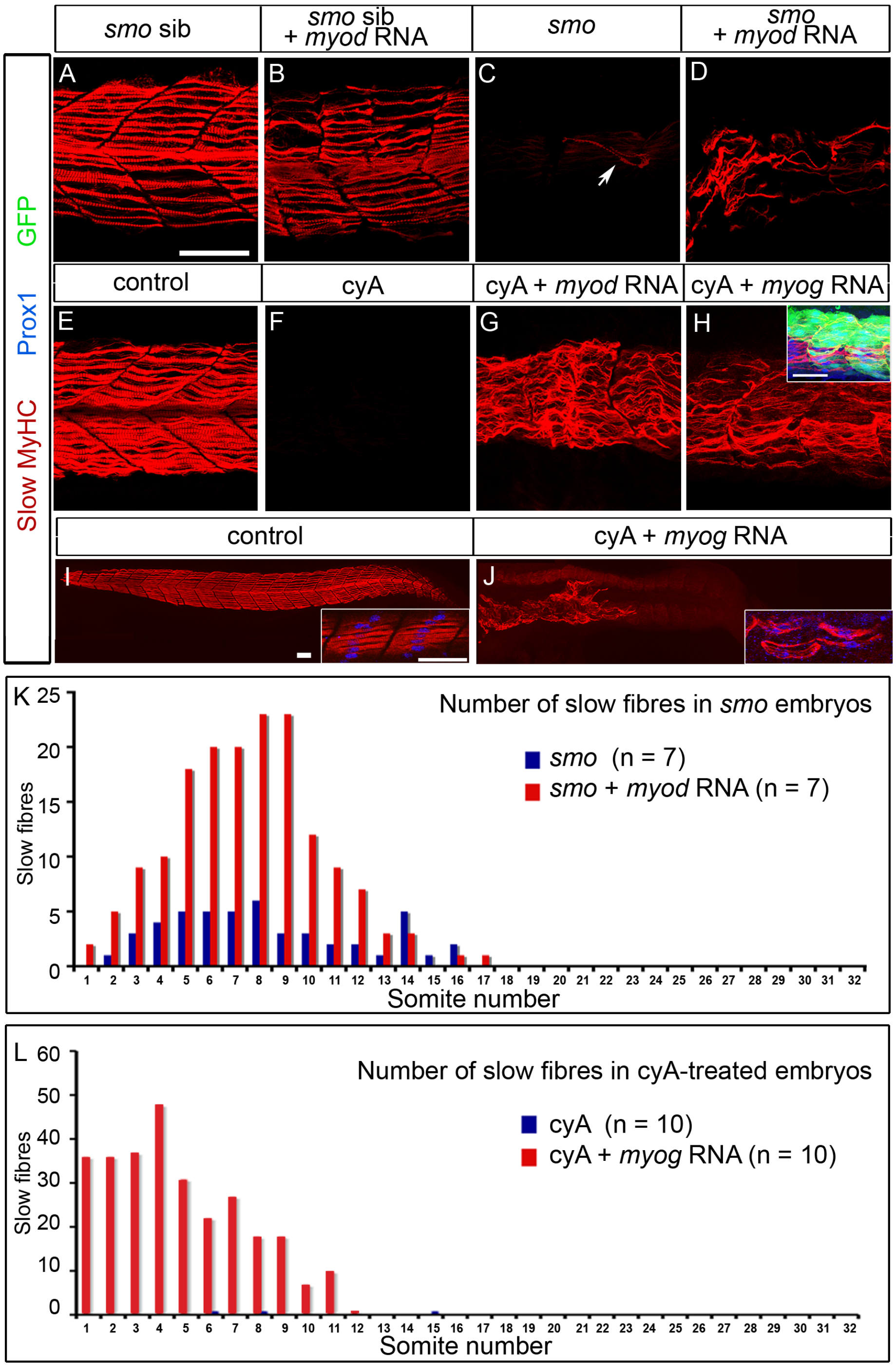

Fig. S3. MRF over-expression rescues trunk slow myogenesis.
Confocal stacks showing immunodetection of slow fibres with Slow MyHC in *smo* mutant (identified by lack of tail circulation), *smo* sibling, un-injected control or cyA-treated embryos injected with *myod* or *myog* RNA. All embryos orientated anterior to left dorsal up showing 2-3 trunk somites (A-H) or entire trunk and tail (I,J). **A,B.** *Myod* RNA-injected *smo* siblings have slow muscle with disrupted somite morphology. **C,D.** Rare slow fibres present in the trunk region of *smo* mutants (arrow) are more common after *myod* RNA injection. **E,F.** CyA-treatment prevents slow fibre formation. Presence of maternal Smo protein may account for the greater number of residual slow fibres in *smo* mutant compared to cyA-treated embryos. **G,H.** *Myod* or *myog* RNA injection rescues slow fibre formation in cyA-treated embryos. Inset in H shows co-expression of slow MyHC, Prox1 and GFP in a cyA-treated embryo after injection of *myog-IRES-GFP* RNA. **I,J.** *Myog* RNA rescues slow fibres in trunk but not tail. Insets show co-expression of Prox1 and slow MyHC in short confocal stacks. **K.** Slow fibres were counted at 24 hpf in each somite of seven control *smo* mutants and seven *smo* mutants injected at 1 cell stage with *myod* RNA. **L.** Slow fibres were counted at 24 hpf in each somite of ten control uninjected and ten embryos injected at 1 cell stage with *myog* RNA that were each subsequently treated with cyA from 30% epiboly. Lack of slow fibre induction in tail could reflect either differential tissue sensitivity or dilution of the injected mRNA. Bars: 50 µm.

Development • Supplementary information

Fig. S4

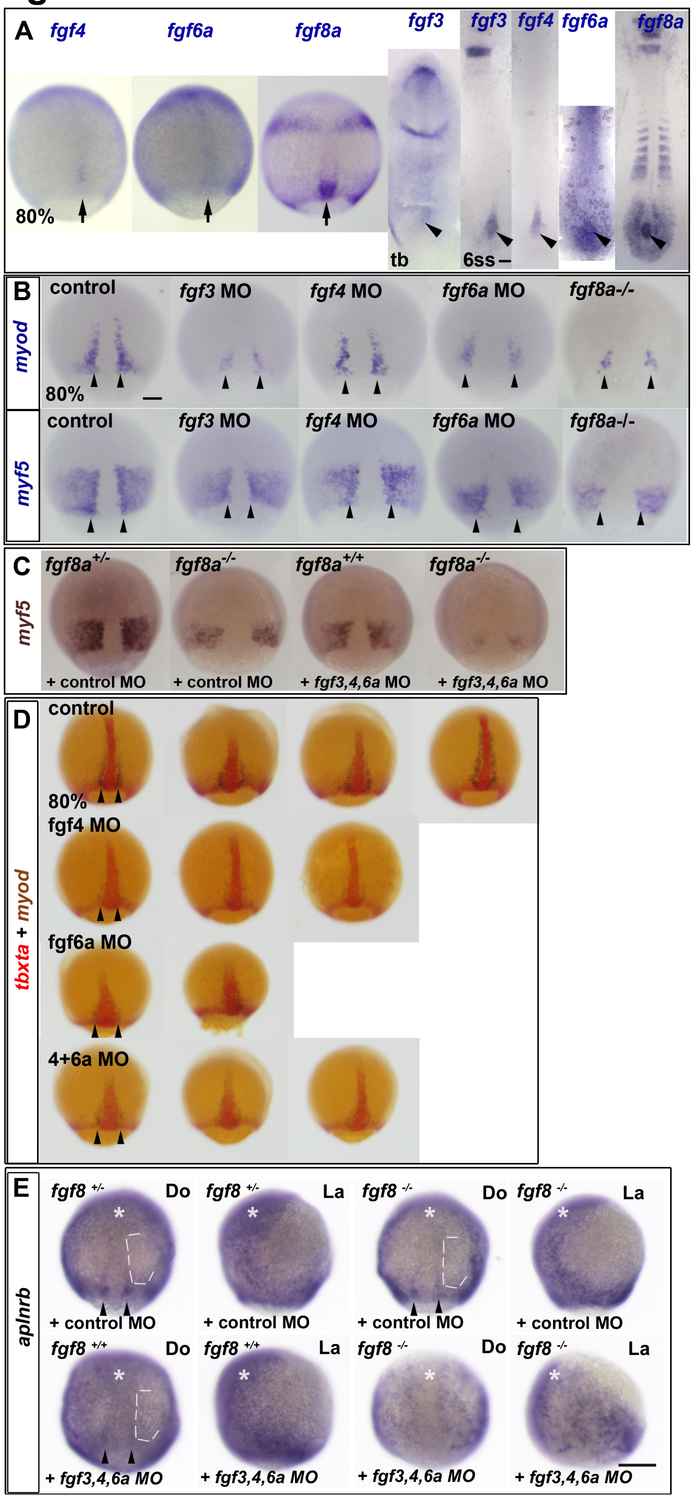

Fig. S4. ***Dorsal fgf expression and requirement for adaxial myogenesis.***
In situ mRNA hybridization for fgfs in wild type embryos at 80% epiboly, tailbud (tb) and 6ss (A), for *myod* and *myf5* in control and fgf MO-injected and *fgf8a*$^{-/-}$ embryos at 80% (B,C) and for *tbxta* (red) and *myod* (blue/brown) (D). **A.** *fgf8a, fgf4*, *fgf6a* and *fgf3* transcripts appear successively in wild type embryos in the dorsal midline (arrows) and CNH (arrowheads). **B.** *Myod* and *myf5* mRNAs in *fgf3* MO, *fgf4* MO and *fgf6a* MO wild type embryos and in sequence-genotyped *fgf8a*$^{-/-}$ embryos at 80% epiboly (upper rows, control and single MOs from a representative experiment). Note that siblings of the *fgf8a* mutants had similar MRF expression. Arrowheads indicate nascent adaxial cells. **C.** *Myf5* mRNA in sibling embryos from an incross of heterozygous *fgf8a*$^{+/-}$ fish injected with 6 ng control MO or 2 ng each of *fgf3*, *fgf4* and *fgf6a* MO. Note the successively stronger reduction in signal as more fgf function is removed. **D.** Rows showing replicate fgf MO-injected embryos had reduced accumulation of *myod* mRNA in pre-adaxial cells of compared to control (arrowheads). Note widening of notochord in *fgf6a* morphants. **E.** *Aplnrb* mRNA in fgf MO- or control MO-injected embryos from an incross of heterozygous *fgf8a*$^{+/-}$ fish showing good gastrulation (asterisks), but reduction in *aplrnb* mRNA in pre-adaxial cells (arrowheads) and increase in paraxial region (white dashes) in dorsal (Do) and lateral (La; dorsal to right) view when fgf signalling was reduced (lower right). Bars: 100 μm.

Development • Supplementary information

Fig. S5

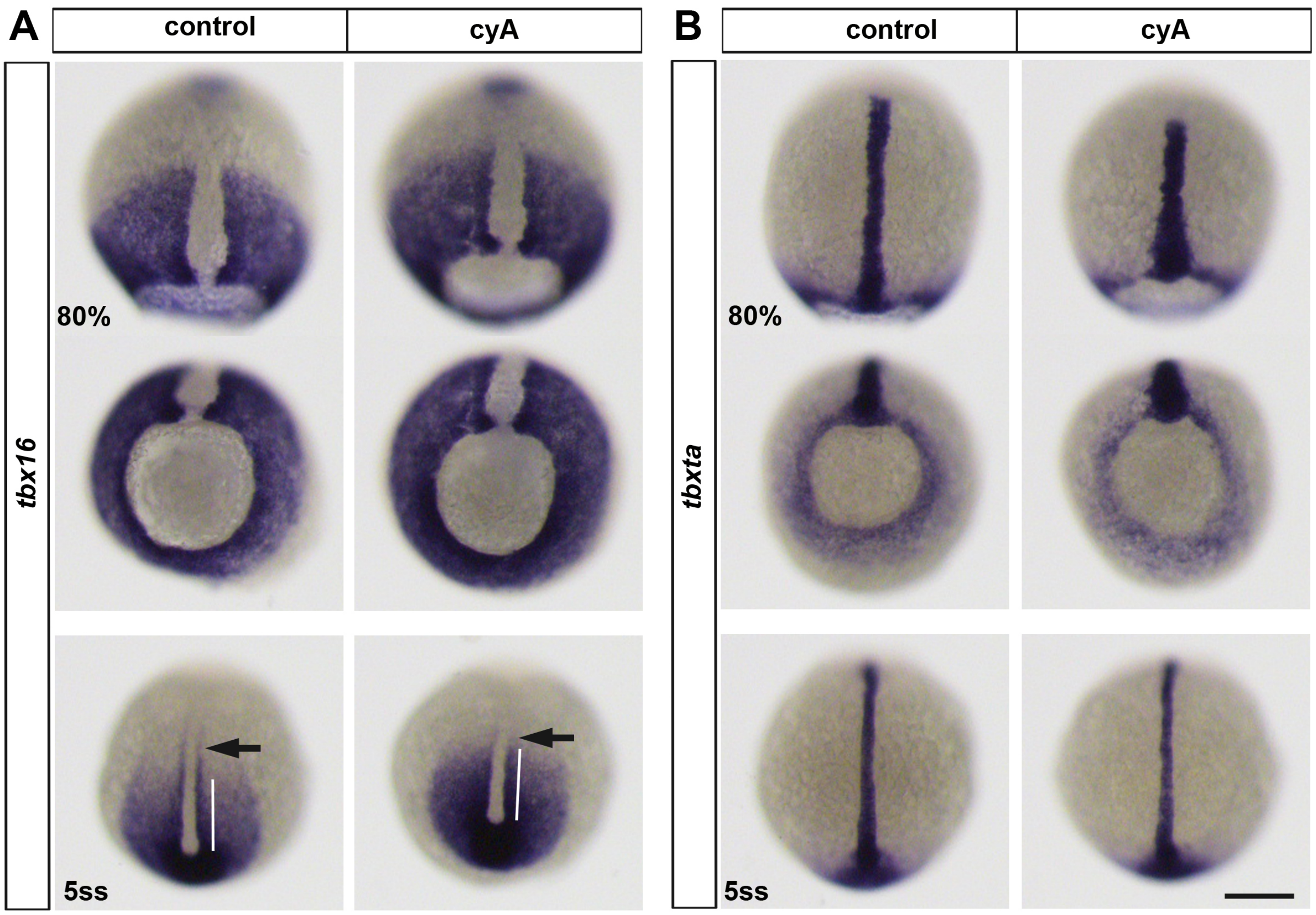

Fig. S5. Tbx mRNA changes in response to Hedgehog signalling blockade.
In situ mRNA hybridization for *tbx16* (A) and *tbxta* (B) in cyA- and control vehicle-treated wild type embryos at 80% epiboly and 5 ss developmental stages. Single embryos are shown from dorsal (80% and 5ss) and vegetal (80%) views. Adaxial *tbx16* mRNA is unaffected by cyA treatment as cells leave the germ ring/tailbud (white line), but is diminished in anterior PSM (arrowheads). Bar: 200 μm.

Development • Supplementary information

Fig. S6

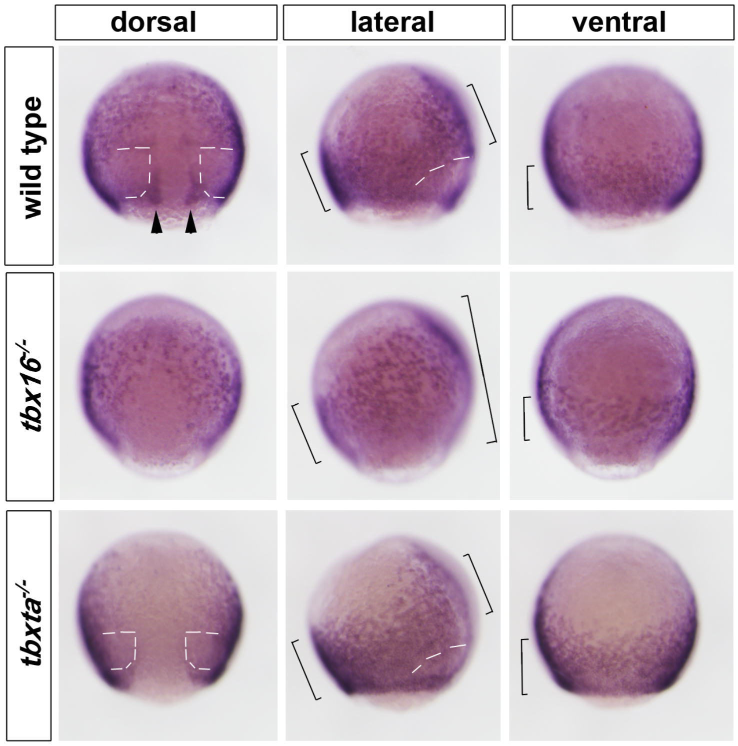

Fig. S6. *Aplnrb* mRNA changes in Tbx mutants.

In situ mRNA hybridization for *aplnrb* in wild type sibling and *tbx16* mutant and *tbxta* mutant embryos at 80% epiboly. Single embryos are shown from dorsal, left lateral and ventral views. Labelling (brackets) is in a band of anterior mesoderm. Note the unlabelled region in wild type and *tbxta* mutant that is missing in *tbx16* mutant (white dashes). Adaxial *aplnrb* mRNA up-regulation (arrowheads) is lacking in mutants.

Development • Supplementary information

Fig. S7

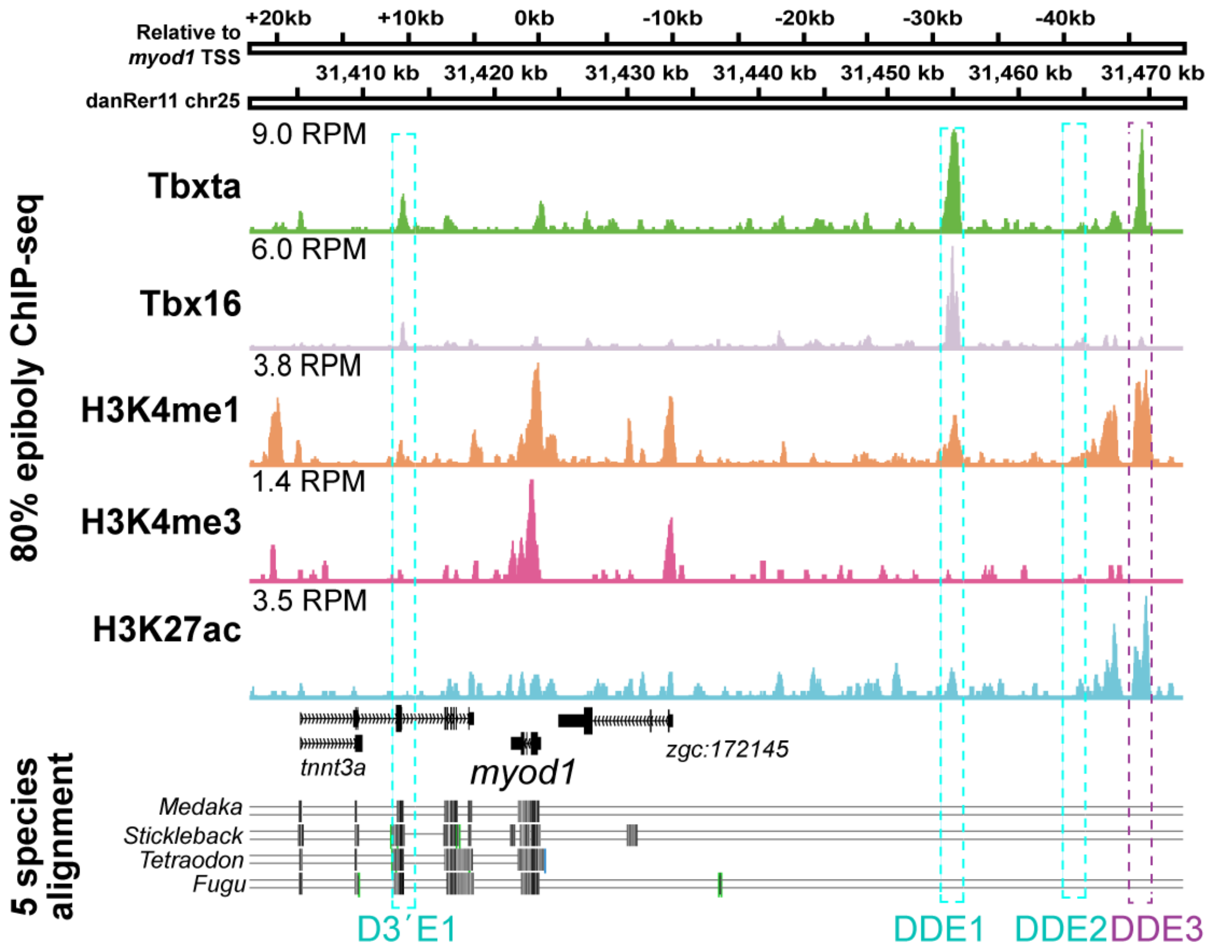

Fig. S7. ChIP-seq analysis of *myod* locus.
ChIP-seq on wt embryos at 75-85% epiboly indicates endogenous Tbx16 and Tbxta binding events within 75 kb flanking *myod* TSS. H3K4me3 marks TSSs; H3K4me1 marks enhancers; H3K27ac indicates active enhancers; RPM – ChIP-seq peaks height in reads per million reads. Multiz Alignments & Conservation from UCSC Genome Browser (Haeussler et al., 2019) are shown beneath. Purple boxes indicate significant Tbx binding for Tbx16 and Tbxta (DDE1) and Tbxta alone (DDE3). Cyan boxes indicate of Tbx sites mentioned in text. Significant H3K4me1 marks are present at both DDE1 and DDE3, while only DDE3 has a significant H3K27ac mark.

Development • Supplementary information

Fig. S8

A. Approximate regional division of myogenesis

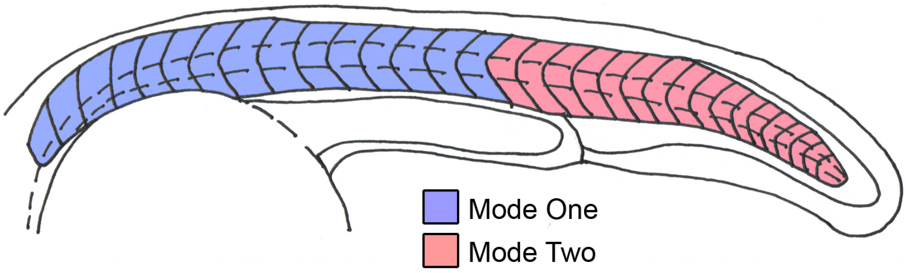

B. Mode One: Trunk slow myogenesis

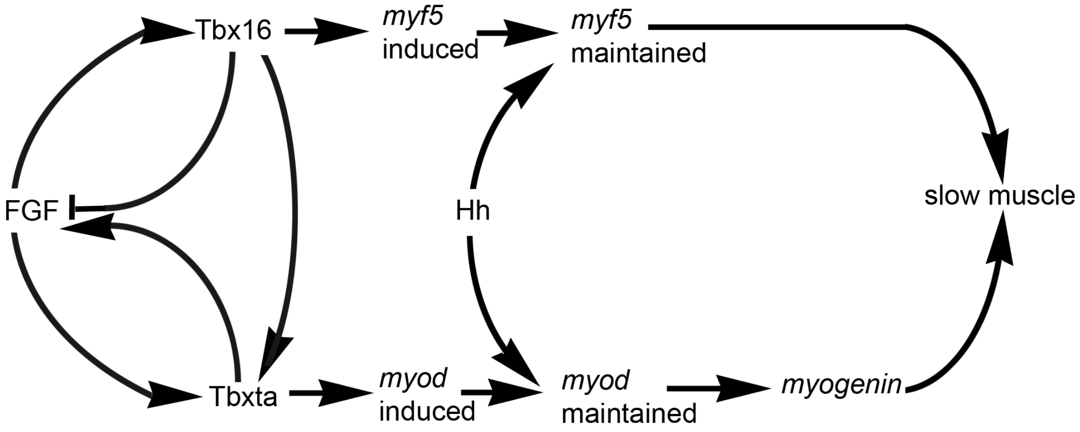

C. Mode Two: Tail slow myogenesis

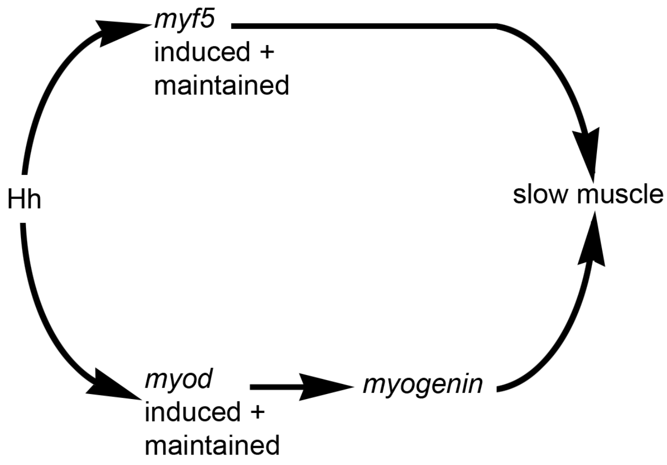

Fig. S8. Model illustrating the diminishing role of Fgf signalling in slow myogenesis from trunk to tail. Note that not all interactions in precursors of adaxial slow muscle cells are shown. In particular, our data indicate that Fgf has effects on both the accumulation of Tbx mRNAs and subsequently on the activity of Tbx proteins. Note also that Fgf-dependent Tbx16 regulation of *myf5* mRNA accumulation in paraxial precursors of fast muscle and dermomyotome is not illustrated.

Development • Supplementary information

Table S1 Quantitation of data in Figures

Figure + panel	Assay	Treatment/genotype	Embryos with phenotype shown/Total (%)
1A	*myod* mRNA	control<br>SU5402<br>control<br>cyA	51/51 (100%)<br>40/40 (100%)<br>80/80 (100%)<br>60/60 (100%)
1A	*myf5* mRNA	control<br>SU5402<br>control<br>cyA	50/50 (100%)<br>36/36 (100%)<br>81/81 (100%)<br>68/68 (100%)
1A	*aplnrb* mRNA	control<br>SU5402	30/30 (100%)<br>28/28 (100%)
1B	*myod* mRNA	cyA 6ss<br>*smo* 6ss<br>*smo* + cyA 6ss<br>*smo* 15ss	83/83 (100%)<br>13/46 (28%)<br>14/54 (26%)<br>26/99 (26%)
1B	*ptc1* mRNA	*smo* 6ss<br>cyA	11/67 (16%)<br>31/31 (100%)
2A	Slow MyHC	*shha* 24hpf	23/80 (29%)
2B	Slow MyHC	*noto* 24hpf	25/88 (28%)
2C	*myod* mRNA	sib<br>*noto*<br>sib + cyA<br>*noto* + cyA	47/62 (76%)<br>15/62 (24%)<br>39/56 (70%)<br>17/56 (30%)
2D	*myod* mRNA	control<br>cyA<br>SU5402<br>SU5402 + cyA	67/67 (100%)<br>47/47 (100%)<br>116/116 (100%)<br>62/63 (98%)
2D	*myf5* mRNA	control<br>cyA<br>SU5402<br>SU5402+cyA	20/20 (100%)<br>15/15 (100%)<br>5/5 (100%)<br>13/13 (100%)
3A	*myod* mRNA	control<br>*fgf6a* MO + *fgf8a* MO<br>*fgf4* MO + *fgf8a* MO<br>*fgf4* MO + *fgf6a* MO + *fgf8a* MO	35/40 (88%) + 8/19 (42%)<br>38/46 (83%) + 22/26 (85%)<br>25/36 (69%)<br>13/15 (87%)
3A	*myf5* mRNA	control<br>*fgf6a* MO + *fgf8a* MO<br>*fgf4* MO + *fgf8a* MO<br>*fgf4* MO + *fgf6a* MO + *fgf8a* MO	38/40 (95%) + 20/20 (100%)<br>46/50 (97%) + 32/33 (91%)<br>11/38 (29%)<br>17/25 (68%)
3B	*myod* mRNA	control<br>cyA<br>fgf4 MO + *fgf8a* MO<br>fgf4 MO + *fgf8a* MO + cyA	3/3 (100%)<br>5/5 (100%)<br>5/5 (100%)<br>4/5 (80%)
3C	*myod* mRNA	control (fgf4)<br>+ *fgf4* mRNA<br>control (fgf6a)<br>+ *fgf6a* mRNA	129/129 (100%)<br>131/136 (96%)<br>20/20 (100%)<br>20/20 (100%)
3C	*myf5* mRNA	control (fgf4)<br>+ *fgf4* mRNA<br>control (fgf6a)<br>+ *fgf6a* mRNA	30/30 (100%)<br>34/40 (85%)<br>20/20 (100%)<br>18/18 (100%)
3D	*aplnrb* mRNA	control<br>+ *fgf4* mRNA	32/32 (100%)<br>12/18 (67%)

**Figure + panel**	**Assay**	**Treatment/genotype**	**Embryos with phenotype shown/Total (%)**
4A	*tbxta* mRNA	control 80%<br>low SU5402 80%<br>control 6ss<br>low SU5402 6ss<br>high SU5402 6ss	21/21 (100%)<br>14/14 (100%)<br>52/52 (100%)<br>44/50 (88%)<br>32/32 (100%)
4A	*tbx16* mRNA	control 80%<br>low SU5402 80%<br>control 6ss<br>low SU5402 6ss<br>high SU5402 6ss	21/21 (100%)<br>15/15 (100%)<br>40/40 (100%)<br>41/41 (100%)<br>74/74 (100%)
4B	*myod* mRNA	control<br>*tbxta* MO<br>*tbx16* MO	28/28 (100%)<br>20/21 (95%)<br>20/22 (91%)
4B	*myf5* mRNA	control<br>*tbxta* MO<br>*tbx16* MO	30/30 (100%)<br>12/19 (63%)<br>19/20 (95%)
4B	*fgf8a* mRNA	control<br>*tbxta* MO<br>*tbx16* MO	30/30 (100%)<br>19/20 (95%)<br>18/20 (90%)
4B	*fgf3* mRNA	control<br>*tbxta* MO<br>*tbx16* MO	25/25 (100%)<br>17/17 (100%)<br>11/14 (79%)
4B	*fgf4* mRNA	control<br>*tbxta* MO<br>*tbx16* MO	28/28 (100%)<br>19/19 (100%)<br>12/14 (86%)
4B	*fgf8a* mRNA	control<br>*tbxta* MO<br>*tbx16* MO	30/30 (100%)<br>14/16 (88%)<br>10/12 (83%)
4C	*myod* mRNA	control<br>*tbxta* MO<br>*tbx16* MO<br>cyA<br>cyA + tbxta MO<br>cyA + tbx16 MO	31/31 (100%)<br>21/22 (95%)<br>24/24 (100%)<br>30/30 (100%)<br>23/23 (100%)<br>22/22 (100%)
4C	*myf5* mRNA	control<br>*tbxta* MO<br>*tbx16* MO<br>cyA<br>cyA + tbxta MO<br>cyA + tbx16 MO	40/40 (100%)<br>24/26 (92%)<br>25/25 (100%)<br>38/38 (100%)<br>19/20 (95%)<br>24/24 (100%)
5A	*myod* mRNA	sib<br>*tbx16-/-*<br>sib + fgf4 mRNA<br>*tbx16-/-* + fgf4 mRNA	82/122 (67%)<br>40/122 (33%)<br>95/128 (78%)<br>33/128 (26%)
5A	*myf5* mRNA	sib<br>*tbx16-/-*<br>sib + fgf4 mRNA<br>*tbx16-/-* + fgf4 mRNA	95/120 (79%)<br>25/120 (21%)<br>98/126 (78%)<br>28/126 (22%)
5B	*tbxta* mRNA	control<br>fgf4 mRNA	30/30 (100%)<br>20/21 (95%)
5B	*tbx16* mRNA	control<br>fgf4 mRNA	29/29 (100%)<br>61/61 (100%)

Development • Supplementary information

Figure + panel	Assay	Treatment/genotype	Embryos with phenotype shown/Total (%)
5C	*myod* mRNA	control<br>+tbx16 mRNA<br>control + 10 μM SU5402<br>+tbx16 mRNA + 10 μM SU5402	31/31 (100%)<br>7/42 (17%)<br>32/32 (100%)<br>7/35 (20%)
5C	*myf5* mRNA	control<br>+tbx16 mRNA<br>control + 10 μM SU5402<br>+tbx16 mRNA + 10 μM SU5402	24/24 (100%)<br>14/42 (33%)<br>29/29 (100%)<br>4/35 (11%)
5D	*myf5* mRNA	control<br>+tbx16 mRNA<br>control + 60 μM SU5402<br>+tbx16 mRNA + 60 μM SU5402	18/18 (100%)<br>2/23 (9%), 21/23 (91%) faint<br>8/8 (100%)<br>23/23 (100%)
5D	*myod* mRNA	control<br>+tbx16 mRNA<br>control + 60 μM SU5402<br>+tbx16 mRNA + 60 μM SU5402	15/15/ (100%)<br>2/24 (8%), 7/24 (29%) disrupted<br>15/15 (100%)<br>32/32 (100%)
6E	*myf5* mRNA	CHD alone<br>CHD + DEX	75/75 (100%)<br>35/70 (50%)
6E	*myod* mRNA	CHD alone<br>CHD + DEX	28/28 (100%)<br>11/21 (52%)
6F	*myod* mRNA	Control *myf5* het incross<br>*myf5+/+* + tbx16 mRNA<br>*myf5+/-* + tbx16 mRNA<br>*myf5-/-* + tbx16 mRNA	33/33 (100%)<br>6/6 (100%)<br>11/17 (65%)<br>2/4 (50%)
7A	*myod* mRNA	sib<br>*tbxta-/-*<br>sib + *fgf4* mRNA<br>*tbxta-/-* + *fgf4* mRNA	100/132 (76%)<br>32/132 (24%)<br>16/27 (59%) (for genotyping see<br>8/27 (29%) Table S4)
7A	*myf5* mRNA	sib<br>*tbxta-/-*<br>sib + *fgf4* mRNA<br>*tbxta-/-* + *fgf4* mRNA	108/138 (78%)<br>30/138 (22%)<br>50/74 (68%)<br>24/74 (32%)
7B	*aplnrb* mRNA	sib<br>*tbxta-/-*<br>sib + *fgf4* mRNA<br>*tbxta-/-* + *fgf4* mRNA	144/195 (74%)<br>51/195 (26%)<br>12/18 (67%)<br>6/18 (33%)
7D	*tbx16* mRNA	sib<br>*tbxta-/-*<br>sib + *fgf4* mRNA<br>*tbxta-/-* + *fgf4* mRNA	48/66 (73%)<br>18/66 (27%)<br>70/93 (75%)<br>23/93 (25%)
8A	Bright field	sib<br>*tbx16-/-*<br>+ *fgf4* mRNA	28/41 (68%)<br>13/41 (32%)<br>48
8B	*myhz1* mRNA	sib<br>*tbx16-/-*<br>sib + *fgf4* mRNA<br>*tbx16-/-* + *fgf4* mRNA	27/40 (68%)<br>13/40 (32%)<br>38/48 (79%)<br>10/48 (21%)

Development • Supplementary information

Figure + panel	Assay	Treatment/genotype	Embryos with phenotype shown/Total (%)
8C,D	*tbx16* mRNA+ Tbxta protein	sib<br>*tbx16-/-*<br>sib + *fgf4* mRNA<br>*tbx16-/-* + *fgf4* mRNA	33/47 (70%)<br>14/47 (30%)<br>40/57 (70%)<br>17/57 (30%)
8E		sib<br>*tbxt16-/-*<br>sib + *fgf4* mRNA<br>*tbx16-/-* + *fgf4* mRNA	19/28 (68%)<br>9/28 (32%)<br>19/31 (61%)<br>12/31 (39%)
S1	*aplnrb* mRNA	50%-10ss	approx. 25 embryos/stage
S2A	Slow MyHC	*smo* sib	27/40 (67.5%)
S2B	Slow MyHC	*smo* sib + *myod* mRNA	28/35 (80%)
S2C	Slow MyHC	*smo*$^{-/-}$	13/40 (32.5%)
S2D	Slow MyHC	*smo*$^{-/-}$ + *myod* mRNA	7/35 (20%)
S2E	Slow MyHC	control	100/100 (100%)
S2F	Slow MyHC	cyA	73/73 (100%)
S2G	Slow MyHC	cyA + *myod* mRNA	20/32 (63%)
S2H	Slow MyHC + Prox1 + GFP	cyA + *myog* mRNA	26/46 (57%)
S2I	Slow MyHC + Prox1	24hpf	50/50 (100%)
S2J	Slow MyHC + Prox1	24hpf	26/43 (60%)
S3B	*myod* mRNA	control<br>+ *fgf3* MO<br>+ *fgf4* MO<br>+ *fgf6a* MO<br>*fgf8a-/-*	39/47<br>58/65<br>46/70<br>52/69
S3B	*myf5* mRNA	control<br>+ *fgf3* MO<br>+ *fgf4* MO<br>+ *fgf6a* MO<br>*fgf8a-/-*	51/51 (100%)<br>21/58 (36%)<br>19/58 (33%)<br>44/69 (64%)
S3C	*myf5* mRNA	sib + control MO<br>*fgf8a-/-* + control MO<br>sib + triple Fgf MO<br>*fgf8a-/-* + triple Fgf MO	13/17 (76%) (2/2 genotyped sib)<br>4/17 (24%) (3/4 genotyped -/-)<br>19/31 (77%) (2/2 genotyped sib)<br>7/31(23%) (3/3 genotyped -/-)
S3D	*myod mRNA*	control<br>+ *fgf4* MO<br>+ *fgf6a* MO<br>+ *fgf4* MO + *fgf6a* MO	4/4<br>3/3<br>2/2<br>3/3
S3E	*aplnrb* mRNA	+ Control MO<br>*fgf8a+/+* + triple Fgf MO<br>*fgf8a-/-* + triple Fgf MO	42/42 (genotyped: 1 +/+, 5 +/-, 2 -/-)<br>32/38 (genotyped: 1 +/+, 3 +/-)<br>6/38 (genotyped: 3/3 -/-)
S4A	*tbx16* mRNA	control @ 80%<br>cyA @ 80%<br>control @ 5ss<br>cyA @ 5ss	16/16<br>19/19<br>33/33<br>18/18
S4B	*tbxta* mRNA	control @ 80%<br>cyA @ 80%<br>control @ 5ss<br>cyA @ 5ss	13/13<br>25/25<br>29/29<br>32/32

Development • Supplementary information

Table S2 Sequences of morpholinos and primers

Morpholino

**Gene**	**Sequence** (start codon underlined)	**Quantity (ng)**	**Reference**
fgf3	5'-CATTGTGGCATGGCGGGATGTCGGC-3'	7.5	(Maroon et al., 2002)
fgf4	5'-GCAAGAGGGCTGAC*T*GGACACTCAT-3'	2-6	
fgf6a	5'-TGAGGAACCTTTGCGCAGTGGCCAT-3'	2-6	
fgf8a	5'-GAGTCTCATGTTTATAGCCTCAGTA -3'	2	(Furthauer et al., 2001)
tbx16	5'-GCTTGAGGTCTCTGATAGCCTGCAT-3'	0.5	(Bisgrove et al., 2005)
tbxta	5'-GACTTGAGGCAG*G*CATATTTCCGAT -3'<br>5'-GCTGGTCGGGACTTGAGGCAGACAT-3'	0.25<br>2	(Bisgrove et al., 2005; Feldman and Stemple, 2001)
control	5'-CCTCCTACCTCAGTTACAATTTATA -3'	3-6	Gene Tools standard

Primers (start and stop codons underlined)

**Gene**	**Forward**	**Reverse**	**Reference**
fgf4	5'-GAGCTCGAGCTCATGAGTGTCC AGTCGGCCCTCTTG-3'	5'-GTCGACGTCGACTCAAATTCTAGGCA AG-3'	
5DE_ChIP-qPCR	5'-TTCCTCACCGTACCTTTTGC-3'	5'-CATTTCCCCCACAATACACC-3'	
5PE1_ChIP-qPCR	5'-GTGCAATTTTGGCTCAGCTT-3'	5'-AGATCGGGGAACTTCGCTAT-3'	
Negative region (*rhod*)	5'-GACTCCACACAATCTGCAACAT-3'	5'-ACCACCTACGCTAAAGAAACCA-3'	Morley et al., 2009

Development • Supplementary information

Table S3 Location and histone modifications of Tbx16 and Tbxta ChIP-seq peaks on *myf5* and *myod* loci

Tbx16 ChIP-seq

***myf5* locus** — Zv9/danRer7 | GRCz11/danRer11

Putative enhancer ID	chr	start	stop	Tbx16 ChIP1 *P* value	Tbx16 ChIP2 *P* value	H3K4me1	H3K4me3	H3K27ac	start	stop	size	Distance from TSS to peak centre
**5DE**	chr4	20596134	20597306	2.36592E-91	3.7325E-52	Yes	No	Yes	21660444	21661616	1173	-80198.5
**5PE1**	chr4	20672749	20673195	5.47016E-11	6.93426E-08	Yes	No	Yes	21737059	21737505	447	-3945.5
**5PE3**	chr4	20680778	20681312	1.69434E-08	1.96789E-06	No	No	No	21745088	21745622	535	+4126.5

***myod* locus**

Putative enhancer ID	chr	start	stop	Tbx16 ChIP1 *P* value	Tbx16 ChIP2 *P* value	H3K4me1	H3K4me3	H3K27ac	start	stop	size	Distance from TSS to peak centre
**D3'E1**	chr25	32256140	32256596	8.74984E-14	2.1727E-06	No	No	No	31412869	31413325	457	+10,396
**DDE1**	chr25	32297466	32298512	3.92645E-30	3.9355E-09	Yes	No	No	31454195	31455241	1047	-31224.5
**DDE2**	chr25	32307295	32307540	1.45546E-05	5.22396E-05	No	No	No	31464024	31464269	246	-40653

Tbxta ChIP-seq

***myf5* locus** — Zv9/danRer7 | GRCz11/danRer11

Putative enhancer ID	chr	start	stop	Tbxta ChIP1 *P* value	Tbxta ChIP2 *P* value	H3K4me1	H3K4me3	H3K27ac	start	stop	size	Distance from TSS to peak centre
**5DE**	chr4	20595996	20597295	N.S.	2.8774E-90	Yes	No	Yes	21660306	21661605	1300	-80273
**5PE2**	chr4	20674923	20675240	4.46684E-06	1.1298E-13	Yes	No	No	21739233	21739550	318	-1837

***myod* locus**

Putative enhancer ID	chr	start	stop	Tbxta ChIP1 *P* value	Tbxta ChIP2 *P* value	H3K4me1	H3K4me3	H3K27ac	start	stop	size	Distance from TSS to peak centre
**DDE1**	chr25	32297085	32298653	N.S.	2.26464E-12	Yes	No	No	31453814	31455382	1569	-31104.5
**DDE3**	chr25	32312015	32312366	1.08643E-07	1.14551E-37	Yes	No	Yes	31468744	31469095	352	-45426

Peaks with significant H3K4me1 and H3K27ac
N.S. = not significant

Table S4. Tbxta dosage controls response of *myod* to Fgf.

Fgf4 mRNA (pg)	Genotype	Number genotyped [%]¶	*Myod* mRNA expression pattern in genotyped embryos (%)		
			Absent	Adaxial only	Expanded ventrally
0	-/-<br>+/-<br>+/+<br>Total	4*<br>3*<br>2*<br>9	4 (100%)§<br>0 (0%)<br>0 (0%)<br>8 ((15%))∞	0 (0%)<br>3 (100%)<br>2 (100%)<br>45 ((85%))	0 (0%)<br>0 (0%)<br>0 (0%)<br>0 ((0%))
100	-/-<br>+/-<br>+/+<br>Total	8 [30%]<br>14 [52%]<br>5 [19%]<br>27	7 (88%)<br>0 (0%)<br>0 (0%)<br>7 (26%)	1 (12%)<br>11 (79%)<br>0 (0%)<br>12 (44%)	0 (0%)<br>3 (21%)<br>5 (100%)<br>8 (30%)
0	-/-<br>+/-<br>+/+<br>Total	5*<br>2*<br>3*<br>10	5 (100%)<br>0 (0%)<br>0 (0%)<br>9 ((30%))	0 (0%)<br>2 (100%)<br>3 (100%)<br>21 ((70%))	0 (0%)<br>0 (0%)<br>0 (0%)<br>0 ((0%))
100	-/-<br>+/-<br>+/+<br>Total	1 [6%]<br>8 [50%]<br>7 [44%]<br>16	1 (100%)<br>0 (0%)<br>0 (0%)<br>1 (6%)	0 (0%)<br>3 (37%)<br>1 (14%)<br>4 (25%)	0 (0%)<br>5 (63%)<br>6 (86%)<br>11 (69%)
150	-/-<br>+/-<br>+/+<br>Total	6 [25%]<br>9 [38%]<br>9 [38%]<br>24	5 (100%)<br>0 (0%)<br>0 (0%)<br>5 (21%)	1 (17%)<br>6 (67%)<br>2 (22%)<br>9 (38%)	0 (0%)<br>3 (33%)<br>7 (78%)<br>10 (42%)
225	-/-<br>+/-<br>+/+<br>Total	15 [24%]<br>26 [42%]<br>21 [34%]<br>62	12 (80%)<br>0 (0%)<br>0 (0%)<br>12 (19%)	3 (20%)<br>11 (42%)<br>4 (19%)<br>18 (29%)	0 (0%)<br>15 (58%)<br>17 (81%)<br>32 (52%)
**Summary**					
0	-/-<br>+/-<br>+/+<br>Total	9*<br>5*<br>5*<br>19	9 (100%)<br>0 (0%)<br>0 (0%)<br>17 ((20%))	0 (0%)<br>5 (100%)<br>5 (100%)<br>66 ((80%))	0 (0%)<br>0 (0%)<br>0 (0%)<br>0 ((0%))
100-225	-/-<br>+/-<br>+/+<br>Total	30 [23%]<br>57 [44%]<br>42 [33%]<br>129	25 (88%)<br>0 (0%)<br>0 (0%)<br>25 (19%)	5 (12%)<br>31 (54%)<br>7 (17%)<br>43 (33%)	0 (0%)<br>26 (46%)<br>35 (83%)<br>61 (47%)

* Only a subset of control embryos were genotyped (to ensure reproducibility).

§ Percentages in curved brackets represent fraction of embryos of indicated genotype showing listed *myod* expression pattern.

¶ Percentages in square brackets represent fraction of embryos in sample with each genotype.

∞ Percentages in double brackets represent fraction of embryos in sample showing listed *myod* expression pattern.

Development • Supplementary information

Table S5 Summary of Results

Stage (hpf)	Genetic Background	Treatment	Gene product	preadaxial cells	adaxial cells	paraxial mesoderm	somites	marginal zone	germ ring	notochord	Tailbud	slow muscle trunk	slow muscle tail	skeletal muscle	Dermomyotome/connective tissue	figure reference
80-90% Epiboly	wt	none	*myf5*	✓		✓										Fig. 1A
	wt	cyA	*myf5*	✓		✓										Fig. 1A
	wt	60 uM SU5402	*myf5*	✗		✗										Fig. 1A
	wt	fgf3MO	*myf5*	✓		✓										Fig. S3B
	wt	fgf4MO	*myf5*	✓		✓										Fig. S3B
	wt	fgf6aMO	*myf5*	✓		✓										Fig. S3B
	fgf8a-/-	none	*myf5*	✓		✓										Fig. S3B
	wt	fgf6a, fgf8a MO	*myf5*	✗		↓										Fig. 3A
	wt	fgf4, fgf8a MO	*myf5*	✗		↓↓										Fig. 3A
	wt	fgf4, fgf6, fgf8a MO	*myf5*	✗		↓↓										Fig. 3A
	fgf8a-/-	fgf3,fgf4,fgf6a MO	*myf5*	✗		✗										Fig. S3C
	wt	fgf4 mRNA	*myf5*	✔		✓		✓	✓							Fig. 3C
	wt	fgf6a mRNA	*myf5*	✓		✓		✓	✓							Fig. 3C
	wt	tbxtaMO	*myf5*	✗		✓										Fig. 4B
	wt	tbx16MO	*myf5*	✗		↓↓										Fig. 4B
	tbx16-/-	none	*myf5*	✗		✗										Fig. 5A
	tbx16-/-	fgf4 mRNA	*myf5*	✗		✗										Fig. 5A
	wt	tbx16 mRNA	*myf5*	✔		✓		✓	✓							Fig. 5C
	wt	10uM SU5402	*myf5*	↓↓		↓↓										Fig. 5C
	wt	10uM SU5402, *tbx16* mRNA	*myf5*	✓		✓										Fig. 5C
	wt	60uM SU5402, *tbx16* mRNA	*myf5*	✗		✗										Fig. 5D
	wt	*tbx16-GR* mRNA, CHD	*myf5*	✔		✓										Fig. 6E
	wt	*tbx16-GR* mRNA, CHD, DEX	*myf5*	✓		✓			✓							Fig. 6E
	tbxta-/-	none	*myf5*	✓		✓										Fig. 7A
	tbxta-/-	*fgf4* mRNA	*myf5*	✓		✓		✓	✓							Fig. 7A
	wt	none	*myod*	✓												Fig. 1A
	wt	cyA	*myod*	✓												Fig. 1A
	wt	60uM SU5402	*myod*	✗												Fig. 1A
	wt	fgf3MO	*myod*	✓												Fig. S3B
	wt	fgf4MO	*myod*	✓												Fig. S3B
	wt	fgf6aMO	*myod*	✓												Fig. S3B
	fgf8a-/-	none	*myod*	✓												Fig. S3B
	wt	fgf6a, fgf8a MO	*myod*	↓												Fig. 3A
	wt	fgf4, fgf8a MO	*myod*	↓↓												Fig. 3A
	wt	fgf4,fgf6,fgf8a MO	*myod*	✗												Fig. 3A
	wt	fgf4 mRNA	*myod*	✓		✓			✓							Fig. 3C
	wt	fgf6a mRNA	*myod*	✔		✓		✓	✓							Fig. 3C
	wt	tbxtaMO	*myod*	✗												Fig. 4B
	wt	tbx16MO	*myod*	✗												Fig. 4B
	tbx16-/-	none	*myod*	✗												Fig. 5A
	tbx16-/-	*fgf4* mRNA	*myod*	✗												Fig. 5A
	wt	*tbx16* mRNA	*myod*	✔		✓			✓							Fig. 5C
	wt	10uM SU5402	*myod*	↓↓												Fig. 5C
	wt	10uM SU5402, tbx16 mRNA	*myod*	✔												Fig. 5C
	wt	60uM SU5402, tbx16 mRNA	*myod*	✗												Fig. 5D
	wt	*tbx16-GR* mRNA, CHD	*myod*	✓												Fig. 6E
	wt	*tbx16-GR* mRNA, CHD, DEX	*myod*	✔		✓			✓							Fig. 6E
	myf5 +/-	none	*myod*	✓												Fig. 6F
	myf5 +/-	*tbx16* mRNA	*myod*	✓		✓			✓							Fig. 6F
	myf5 -/-	*tbx16* mRNA	*myod*	✓		✓			✓							Fig. 6F
	tbxta-/-	none	*myod*	✗												Fig. 7A
	tbxta-/-	*fgf4* mRNA	*myod*	✗												Fig. 7A
	wt	none	*aplnrb*	✓		✗		✓	✓							Fig. 1A

	wt	SU5402	*aplnrb*	✗		↑		✓	✓							Fig. 1A
	wt	*fgf4* mRNA	*aplnrb*	✓		✗		↓	✓							Fig. 3D
	tbx16-/-	none	*aplnrb*	✗		↑		✓	✓							Fig. S5
	tbxta-/-	none	*aplnrb*	✗		✗		✓	✓							Fig. S5
	tbxta-/-	*fgf4* mRNA	*aplnrb*	✓		✓		↓	✓							Fig. 7B
	fgf8a+/-	control MO	*aplnrb*	✓		✗		✓	✓							Fig. S3E
	fgf8a-/-	control MO	*aplnrb*	✓		✗		✓	✓							Fig. S3E
	fgf8a+/-	fgf3,fgf4,fgf6a MO	*aplnrb*	↓		✗		✓	✓							Fig. S3E
	fgf8a-/-	fgf3,fgf4,fgf6a MO	*aplnrb*	✗		✗		↓	↓							Fig. S3E
	wt	none	*tbxta*						✓	✓						Fig. 4A
	wt	10um SU5402	*tbxta*						↓	↓						Fig. 4A
	wt	*fgf4* mRNA	*tbxta*						↓	↔						Fig. 5B
	wt	cyA	*tbxta*						✓	↔						Fig. S4
	wt	none	*tbx16*	✓				✓	✓							Fig. 4A
	wt	10um SU5402	*tbx16*	✗				↓	↓							Fig. 4A
	wt	*fgf4* mRNA	*tbx16*	↓				✓	↓							Fig. 5B
	wt	cyA	*tbx16*	✓				✓	✓							Fig. S4
	tbxta-/-	none	*tbx16*	↓				✓	✓							Fig. 7D
	tbxta-/-	*fgf4* mRNA	*tbx16*	✗				↓	↓							Fig. 7D
	tbx16-/-	none	*tbx16*	↓↓				↓↓	↓							Fig. 8C
	tbx16-/-	*fgf4* mRNA	*tbx16*	✗				✗	✗							Fig. 8C
	wt	none	*fgf8a*						✓	✓						Fig. 4B
	wt	tbxtaMO	*fgf8a*						✓	✗						Fig. 4B
	wt	tbx16MO	*fgf8a*						↑	✗						Fig. 4B
	tbx16 sib	none	Tbxt						✓	✓						Fig. 8D
	tbx16-/-	none	Tbxt						✓	↔						Fig. 8D
	tbx16 sib	*fgf4* mRNA	Tbxt						✓	↔						Fig. 8D
	tbx16-/-	*fgf4* mRNA	Tbxt			✓		✓	✓	↔						Fig. 8D
	tbx16 sib	none	*shha*							✓						Fig. 8E
	tbx16-/-	none	*shha*							↔						Fig. 8E
	tbx16 sib	*fgf4* mRNA	*shha*			✓				↔						Fig. 8E
	tbx16-/-	*fgf4* mRNA	*shha*	✓	✓	✓		✓	✓	↔						Fig. 8E
Tailbud	wt	none	*myod*	✓	✓											Fig. 3B
	wt	cyA	*myod*	✓	✗											Fig. 3B
	wt	fgf4, fgf8a MO	*myod*	✓	✓											Fig. 3B
	wt	cyA, fgf4, fgf8a MO	*myod*	✗	✗											Fig. 3B
6 somites	wt	none	*myod*	✓	✓		✓									Fig. 1B
	wt	cyA	*myod*	✓	✗		✓									Fig. 1B
	smo-/-	none	*myod*	✓	✗		✓									Fig. 1B
	smo-/-	cyA	*myod*	✓	✗		✓									Fig. 1B
	noto-/-	none	*myod*	✓	✓		✓									Fig. 2C
	noto-/-	cyA	*myod*	✓	✗		✓									Fig. 2C
	wt	tbx16MO*	*myod*	↓	↓		↓↓									Fig. 4C
	wt	tbx16MO*, cyA	*myod*	✗	✗		↓↓									Fig. 4C
	wt	tbxta	*myod*	✗	✓		✓									Fig. 4C
	wt	tbxta, cyA	*myod*	✗	✗		✓									Fig. 4C
	wt	none	*myf5*	✓	✓	✓	✓				✗					Fig. 4C
	wt	tbx16MO*	*myf5*	↓	↓	✓	✓				✗					Fig. 4C
	wt	tbx16MO*, cyA	*myf5*	✗	✗	✓	✓				✗					Fig. 4C
	wt	tbxta	*myf5*	↓	↓	✓	✓				✓					Fig. 4C
	wt	tbxta, cyA	*myf5*	✗	✗	✓	✓				✓					Fig. 4C
	wt	none	*tbxta*							✓	✓					Fig. 4A
	wt	10um SU5402	*tbxta*							✓	↓					Fig. 4A
	wt	30um SU5402	*tbxta*							✗	✗					Fig. 4A
	wt	cyA	*tbxta*							✓	✓					Fig. S4
	wt	none	*tbx16*	✓		✓					✓					Fig. 4A
	wt	10um SU5402	*tbx16*	✓		✓					↓					Fig. 4A

	wt	30um SU5402	*tbx16*	✗		✗					✗					Fig. 4A
	wt	cyA	*tbx16*	↓		✓					✓					Fig. S4
	wt	none	*fgf3*							✓						Fig. 4B
	wt	tbxtaMO	*fgf3*							✗						Fig. 4B
	wt	tbx16MO	*fgf3*							↑	↑					Fig. 4B
	wt	none	*fgf4*							✓						Fig. 4B
	wt	tbxtaMO	*fgf4*							✗						Fig. 4B
	wt	tbx16MO	*fgf4*							↑						Fig. 4B
	wt	none	*fgf8a*			✓	✓			✓	✓					Fig. 4B
	wt	tbxtaMO	*fgf8a*			✓	✓			↓	✓					Fig. 4B
	wt	tbx16MO	*fgf8a*			↑	✓			↑	↑					Fig. 4B
8 somites	wt	none	*myod*	✓	✓		✓									Fig. 2D
	wt	cyA	*myod*	✓	✗		✓									Fig. 2D
	wt	SU5402	*myod*	✓	✓		↓									Fig. 2D
	wt	cyA, SU5402	*myod*	✗	✗		↓									Fig. 2D
	wt	none	*myf5*	✓	✓	✓	✓									Fig. 2D
	wt	cyA	*myf5*	✓	✗	✓	✓									Fig. 2D
	wt	SU5402	*myf5*	✓	✓	✓	✓									Fig. 2D
	wt	cyA, SU5402	*myf5*	✗	✗	↓	✓									Fig. 2D
15 somites	*smo* sib	none	*myod*	✓	✓		✓									Fig. 1B
	smo-/-	none	*myod*	✗	✗		✓									Fig. 1B
24 hpf	*shha* sib	none	slow MyHC									✓	✓	✓		Fig. 2A
	shha -/-	none	slow MyHC									✓	✗	↓		Fig. 2A
	noto sib	none	slow MyHC									✓	✓	✓		Fig. 2B
	noto-/-	none	slow MyHC									✓	✗	↓		Fig. 2B
	smo sib	none	slow MyHC									✓	✓	✓		Fig. S2
	smo sib	myod RNA	slow MyHC									✓	✓	↑		Fig. S2
	smo-/-	none	slow MyHC									↓↓	✗	↓		Fig. S2
	smo-/-	myod RNA	slow MyHC									✓	✗	↓		Fig. S2
	wt	cyA	slow MyHC									✗	✗	↓		Fig. S2
	smo-/-	cyA	slow MyHC									✓	✗	↓		Fig. S2
	tbx16 sib	none	*col1a2*												✓	Fig. 8B
	tbx16 sib	*fgf4* mRNA	*col1a2*												↓↓	Fig. 8B
	tbx16 -/-	none	*col1a2*												↓	Fig. 8B
	tbx16 -/-	*fgf4* mRNA	*col1a2*												↓↓	Fig. 8B
	tbx16 sib	none	*myhz1*									✓	✓	✓		Fig. 8B
	tbx16 sib	*fgf4* mRNA	*myhz1*									↓↓	↓↓	↓↓		Fig. 8B
	tbx16 -/-	none	*myhz1*									↓	↓	↓		Fig. 8B
	tbx16 -/-	*fgf4* mRNA	*myhz1*									✗	✗	✗		Fig. 8B

✓	present at normal levels
✗	absent
↓	downregulated
↓↓	severely downregulated, still present
↑	upregulated
↔	mild expansion
↔	moderate expansion
↔	severe expansion

Development • Supplementary information

Bisgrove, B. W., Snarr, B. S., Emrazian, A. and Yost, H. J. (2005). Polaris and Polycystin-2 in dorsal forerunner cells and Kupffer's vesicle are required for specification of the zebrafish left-right axis. *Dev Biol* **287**, 274-288.

Feldman, B. and Stemple, D. L. (2001). Morpholino phenocopies of sqt, oep, and ntl mutations. *Genesis* **30**, 175-177.

Furthauer, M., Reifers, F., Brand, M., Thisse, B. and Thisse, C. (2001). sprouty4 acts in vivo as a feedback-induced antagonist of FGF signaling in zebrafish. *Development* **128**, 2175-2186.

Haeussler, M., Zweig, A. S., Tyner, C., Speir, M. L., Rosenbloom, K. R., Raney, B. J., Lee, C. M., Lee, B. T., Hinrichs, A. S., Gonzalez, J. N., et al. (2019). The UCSC Genome Browser database: 2019 update. *Nucleic Acids Res* **47**, D853-D858.

Maroon, H., Walshe, J., Mahmood, R., Kiefer, P., Dickson, C. and Mason, I. (2002). Fgf3 and Fgf8 are required together for formation of the otic placode and vesicle. *Development* **129**, 2099-2108.
