# Supplementary material for: Fgf-driven Tbx protein activities directly induce myf5 and myod to initiate zebrafish myogenesis
Source: Development. 2020 Apr 28;147(8):dev184689. doi: 10.1242/dev.184689 (PMC7197714; doi:10.1242/dev.184689)
Supplement: Reviewer comments [file develop-147-184689_review_history.pdf]

Fgf-driven Tbx protein activities directly induce *myf5* and *myod* to initiate zebrafish myogenesis

Daniel P.S. Osborn, Kuoyu Li, Stephen J. Cutty, Andrew C. Nelson, Fiona C. Wardle, Yaniv Hinits and Simon M. Hughes

DOI: 10.1242/dev.184689

Editor: Stephen Wilson

Review timeline

Original submission: 13 September 2019
Editorial decision: 29 October 2019
First revision received: 29 January 2020
Accepted: 14 February 2020

Original submission

First decision letter

MS ID#: DEVELOP/2019/184689

MS TITLE: Fgf-driven Tbx protein activities directly induce myf5 and myod to initiate zebrafish myogenesis

AUTHORS: Daniel P.S. Osborn, Kuoyu Li, Stephen J Cutty, Andrew C Nelson, Fiona C Wardle, Yaniv Hinits, and Simon M Hughes

I have now received all the referees' reports on the above manuscript, and have reached a decision. The referees' comments are appended below, or you can access them online: please go to BenchPressand click on the 'Manuscripts with Decisions' queue in the Author Area.

As you will see, the referees express considerable interest in your work, but have some criticisms and suggestions for improving your manuscript. If you are able to revise the manuscript along the lines suggested, I will be happy receive a revised version of the manuscript. Please also note that Development will normally permit only one round of major revision.

Please attend to all of the reviewers' comments and ensure that you clearly highlight all changes made in the revised manuscript. Please avoid using 'Tracked changes' in Word files as these are lost in PDF conversion. I should be grateful if you would also provide a point-by-point response detailing how you have dealt with the points raised by the reviewers in the 'Response to Reviewers' box. If you do not agree with any of their criticisms or suggestions please explain clearly why this is so.

Reviewer 1

Advance summary and potential significance to field

Osborn and colleagues have investigated the upstream events leading to myogenic activation in zebrafish. In a data rich and well written study the authors identify FGF signaling and T-box transcription factors as operating upstream of early myf5 and myod expression, with interesting differential inputs on activation of the two MRF genes. The work is clearly presented and furthers

© 2020. Published by The Company of Biologists under the terms of the Creative Commons Attribution License (https://creativecommons.org/licenses/by/4.0/).

our understanding of the onset of vertebrate myogenesis with potentially important evolutionary implications.

Comments for the author

The following points should be addressed.

1. Addition of a Table summarizing the results of different treatments (pharmacological and genetic) on MRF expression and different downstream myogenic programs would be very helpful. Similarly, a schema showing the different inputs upstream of myf5 and myod as defined in this study would also be useful for the reader.

2. In the abstract the authors imply a role for tbx16 in commitment and tbxta in differentiation. Do they demarcate these steps by differences between myf5 and myod function? This appears to oversimplify the authors' findings following t-box gene knockdown. Please clarify.

3. Did the authors investigate the effects of knocking down both tbxta and tbx16?

4. In the discussion it would be useful to use the term "feed-forward" as FGF signaling appears to be required for T-box gene expression as well as cooperating with these genes to activate MRF transcription.

5. Please conclude more clearly about the role of myf5 in the activation of myod through tbx16 (page 12). This would appear to reinforce the feed-forward circuitry driving the onset of myogenesis.

6. Can the authors clarify whether the differences observed in tbxta and tbx16 binding are due to divergent target sites or flanking site context?

7. Concerning the discussion of the timing of txbta binding to the myod locus, the authors should consider a role of T-box genes in priming MRF loci for later activation.

8. Concerning the evolutionary point, do the authors conclude that the t-box genes required for FGF driven MRF activation are not involved in Hh regulation of MRF expression? Is expression of tbxta and tbx16 altered after CyA treatment?

9. Can the authors provide data or at least discuss whether the circuitry they uncover upstream of myf5 and myod expression is shared during cranial myogenesis. They mention that cranial mesoderm is resistant to FGF driven myf5 and myod activation - does this include the cells giving rise to branchiomeric muscles?

10. The authors should discuss their findings in the light of the study of myogenic roles of Tbx6 by Nandkishore et al (PMID: 30237317).

<u>Reviewer 2</u>

Advance summary and potential significance to field

This paper describes an analysis of the transcriptional regulation of the MRF genes myf5 and myoD in the developing zebrafish. Despite the fundamental nature of the process of myogenesis, there are surprising gaps in our knowledge about the allocation of mesodermal cells to the skeletal muscle lineage in the fish embryo, which the authors have attempted to address here. The manuscript presents two main bodies of data: gene expression analysis Â– using in situ hybridisation - of genetically and pharmacologically manipulated embryos; and cis-regulation analysis using chromatin immune precipitation.
The first set of experiments confirm and extend previous analyses of the effects of inhibition of FGF and HH signaling on myf5/myod expression, from which the authors conclude that activation of myod in adaxial (slow twitch progenitor) cells requires FGF in the trunk and HH in the tail. This conclusion is well supported by the data. However, it is not clear why injection of myod or myog

© 2020. Published by The Company of Biologists under the terms of the Creative Commons Attribution License (https://creativecommons.org/licenses/by/4.0/).

mRNA into smo or cycA treated embryos should rescue slow myogenesis in the trunk but not the tail (line 163).
To address which FGF ligands mediate the activation MRF activation, the authors have resorted to morpholino oligonucleotide mediated knock down of individual FGFs. The use of this approach, with all its caveats, is somewhat disappointing given the well-established methods for targeted gene inactivation in the zebrafish.
Based on these data, the authors conclude that the combined activity of two FGFs (4 and 8a) is required for the activation of myod and myf5.
The authors next investigated the role of Tbx transcription factors as mediators of the MRF gene regulation by FGF. They show that inhibition of FGF signaling abolishes tbxta and tbx16 expression in trunk mesoderm and, based on morpholino knockdown experiments that Tbx16 is required for the activation of myod in preadaxial cells. This conclusion is supported by the finding that injection of FGF4 mRNA does not induce myod or myf5 expression in tbx16 mutant embryos, whereas injection of tbx16 mRNA can induce both myod and myf5 expression in FGF inhibitor (SU5402) treated embryos.
Taken together, these data provide reasonable support for the authorsÂ' second conclusion (line 444) that Fgf acts through Tbx16 to drive initial myogenic events in the adaxial cell lineage.
Evidence that TBX transcription factors are direct regulators of MRF genes is based on ChIPseq analysis.
These reveal peaks that overlap with histone methylation peaks upstream of the myf5 and myoD genes.
Whilst these data are consistent with the conclusion that Tbx16 activates transcription of myf5 and myod by binding to the elements thus identified, they do not constitute definitive proof of such a role. Mutation of these elements would provide a test of this conclusion.
In summary, this study provides answers to some unresolved issues concerning the regulation of MRF expression and skeletal muscle development in the zebrafish embryo and supports some interesting evolutionary speculation.

Comments for the author

• Figure 1a. I would avoid using "con" as an abbreviation for control in this context as it is also the symbol for the hedgehog pathway mutation "chameleon"
• Line 161. The comment about the effect of loss of shha on trunk versus tail muscle is a bit elliptic. As the authors themselves show (Fig S2), complete loss of HH activity results in loss of muscle from the trunk as well as the tail - so their point (I think) is that the tail is more sensitive to a reduction in Hh activity. This could be made more explicit.
• It would be good to have a control for the specificity of the effects of the Fgf morpholinos – eg. aplnrb expression
• In the cycloheximide experiments, explain how the levels of myoD expression were quantified (line 361)
• The manuscript is quite long and not always an easy read - I appreciate that it describes a complex process but I think some re-writing could improve clarity and make it more accessible to readers who are not totally immersed in the subject.

<u>Reviewer 3</u>

Advance summary and potential significance to field

This manuscript is a tour de force analysis of slow-twitch muscle specification during trunk versus tail development. The data are of high quality and reported appropriately. The work shows that FGF and Tbx 16 cooperate to induce myogenic regulatory factor expression (ChIP-deq data show Tbx16 directly activates myogenic regulatory factors), providing a novel link between mesoderm induction and subsequent specification of muscle. Taken together, this manuscript fills an important hole in our understanding of muscle development.

Comments for the author

Major Comments: My major comments all have to do with the writing/presentation of the manuscript.

© 2020. Published by The Company of Biologists under the terms of the Creative Commons Attribution License (https://creativecommons.org/licenses/by/4.0/).

The manuscript is quite erudite - which in itself is not bad but I suspect there is no chance that a typical graduate student would actually make it through the manuscript and that would be a shame because there is beautiful developmental biology in this manuscript. Some suggestions to help with clarity (it took this reviewer an hour to get through a first read):
1. In the introduction it is not clear when the authors are discussing commonalities between mice and fish and when they are just referencing fish work - please make this more explicit.
Also, it might be useful to including frogs/chickens as appropriate in the Tbx discussion.
2. There are so many genes tested and tools used/different timing of treatments/analysis that it is a slog to get through the manuscript. Suggestions - please write that treating at 30% epiboly in the first results paragraph, please define genes/inhibitors as you use them, and it would be fantastic if the authors could use cartoons throughout the manuscript to highlight their design, rationale, and results.
3. While the discussion about potential evolutionary implications is interesting, I have some concerns. One, I'm not convinced that it is appropriate to state in the abstract that the findings provide insight into ancestral vertebrate trunk myogenesis given how derived zebrafish are... and one would maybe need to do comparative studies in sharks? I would suggest that the authors consider focusing less on evolution in the introduction - where there are some broad statements that are slightly disingenuous (for example, "how might deuterostome muscle have formed prior to evolution of the notochord" - this was confusing to me because invertebrates are perfectly good at generating muscle.... and at least some of the mechanisms appear to be conserved.....). The discussion seems to be a more appropriate place for speculation about evolution of muscle development.
Minor comments:
-The Fgf4 expression is fairly drastic, do the authors have the ability to quickly do more mosaic expression or genetic mosaic analysis to determine whether clusters of Fgf4 expressing cells are sufficient to initiate MRF expression?

First revision

<u>Author response to reviewers' comments</u>

<u>Reviewer 1</u> Advance Summary and Potential Significance to Field:
Osborn and colleagues have investigated the upstream events leading to myogenic activation in zebrafish. In a data rich and well written study the authors identify FGF signaling and T-box transcription factors as operating upstream of early myf5 and myod expression, with interesting differential inputs on activation of the two MRF genes. The work is clearly presented and furthers our understanding of the onset of vertebrate myogenesis with potentially important evolutionary implications.

We are pleased the Reviewer found our study of interest.

Reviewer 1 Comments for the Author: The following points should be addressed.

1. Addition of a Table summarizing the results of different treatments (pharmacological and genetic) on MRF expression and different downstream myogenic programs would be very helpful. Similarly, a schema showing the different inputs upstream of myf5 and myod as defined in this study would also be useful for the reader.

We now provide these as Table S5 and a summary schema (Fig. S7).

2. In the abstract the authors imply a role for tbx16 in commitment and tbxta in differentiation. Do they demarcate these steps by differences between myf5 and myod function? This appears to oversimplify the authors' findings following t-box gene knockdown. Please clarify.

We see the Reviewer's point. We did not intend to imply that Tbxta is only required for differentiation, it simply drives *myod* expression, which is then required for differentiation if

© 2020. Published by The Company of Biologists under the terms of the Creative Commons Attribution License (https://creativecommons.org/licenses/by/4.0/).

Hh signalling is not present (as we showed in Osborn et al 2011). We have changed the Abstract by removing the term 'differentiation' in parallel with the shortening required to conform with the 180 word limit.

3. Did the authors investigate the effects of knocking down both tbxta and tbx16?

No, we did not. But the severe effect of double knockout was reported by Amacher et al 2002, which we discuss on ln 500-507 (all line numbers refer to the pdf entitled *DanKuoyuFgf200128withChanges.pdf*). We presume that complete lack of these Tbx classes (Tbx16, Tbx16l, Tbxta and Tbxtb) would lead to total lack of dorsal mesoderm, so myogenesis could not be analysed.

4. In the discussion it would be useful to use the term "feed-forward" as FGF signaling appears to be required for T-box gene expression as well as cooperating with these genes to activate MRF transcription.

Good point. This is now included on ln 625.

5. Please conclude more clearly about the role of myf5 in the activation of myod through tbx16 (page 12). This would appear to reinforce the feed-forward circuitry driving the onset of myogenesis.

The Reviewer is correct. We have now clarified this issue on ln 398-399.

6. Can the authors clarify whether the differences observed in tbxta and tbx16 binding are due to divergent target sites or flanking site context?

There is no evidence in zebrafish that Tbxta and Tbx16 bind different motifs or that flanking sequences play a role (Garnett et al, 2009, doi: 10.1242/dev/024703; Nelson et al, 2017, doi: 10.1016/j.celrep.2017.06.011). It should be born in mind that the ChIP-seq data presented in this manuscript come from whole embryos, and that Tbxta and Tbx16 are expressed in both overlapping and non-overlapping patterns in the embryo. We showed in Nelson et al (2017) that binding of Tbxta, but not Tbx16, is near genes associated with axial mesoderm/notochord GO terms, where *tbxta*, but not *tbx16*, is expressed. Where we see binding by one factor and not the other this is therefore likely to be due to cell-type specific expression and/or cell type-specific chromatin accessibility, rather than motif specificity.

7. Concerning the discussion of the timing of txbta binding to the myod locus, the authors should consider a role of T-box genes in priming MRF loci for later activation.

The Reviewer is correct. We now include this possibility on ln 616-617.

8. Concerning the evolutionary point, do the authors conclude that the t-box genes required for FGF driven MRF activation are not involved in Hh regulation of MRF expression? Is expression of tbxta and tbx16 altered after CyA treatment?

Good point. We have now done this experiment and show the result in new Fig. S4. Neither *tbxta* nor *tbx16* are altered during gastrulation or around the germ ring/tailbud by CyA treatment. However, the persistence of *tbx16* mRNA in adaxial cells (but not its initial upregulation) is diminished at 5ss and beyond, consistent with the failure of maintenance of the myogenic phenotype, as we published for *myf5* and *myod* mRNAs in Osborn et al 2011. We now mention this at ln 276-277.

9. Can the authors provide data or at least discuss whether the circuitry they uncover upstream of myf5 and myod expression is shared during cranial myogenesis. They mention that cranial mesoderm is resistant to FGF driven myf5 and myod activation - does this include the cells giving rise to branchiomeric muscles?

Yes, we now discuss this on ln 572-577. Unfortunately, our experiments do not address this issue definitively as head muscle forms after 2 dpf and in no experiments did we leave embryos

© 2020. Published by The Company of Biologists under the terms of the Creative Commons Attribution License (https://creativecommons.org/licenses/by/4.0/).

this long. All we can say is what we reported: that extra Fgf fails to induce MRFs in cranial mesoderm at a stage when it does so in trunk mesoderm. Given the gross defects caused later by ectopic Fgf, we do not think further analysis of cranial myogenesis would be meaningful without a local timed manipulation.

10. The authors should discuss their findings in the light of the study of myogenic roles of Tbx6 by Nandkishore et al (PMID: 30237317).

Thanks for pointing us to this embarrassing omission. Our findings seem entirely consistent with the view that zebrafish behave similarly. We now include this reference in Intro on ln 92-94 and discuss this important work on ln 577.

Reviewer 2 Advance Summary and Potential Significance to Field:
This paper describes an analysis of the transcriptional regulation of the MRF genes myf5 and myoD in the developing zebrafish. Despite the fundamental nature of the process of myogenesis, there are surprising gaps in our knowledge about the allocation of mesodermal cells to the skeletal muscle lineage in the fish embryo, which the authors have attempted to address here. The manuscript presents two main bodies of data: gene expression analysis – using in situ hybridisation - of genetically and pharmacologically manipulated embryos; and cis-regulation analysis using chromatin immune precipitation.
The first set of experiments confirm and extend previous analyses of the effects of inhibition of FGF and HH signaling on myf5/myod expression, from which the authors conclude that activation of myod in adaxial (slow twitch progenitor) cells requires FGF in the trunk and HH in the tail. This conclusion is well supported by the data. However, it is not clear why injection of myod or myog mRNA into smo or cycA treated embryos should rescue slow myogenesis in the trunk but not the tail (line 163).

This is a fair point. The answer could be technical (RNA dilution in tailbud-derived cells, say) or functional. We now make this explicit in ln 1343-4 (all line numbers refer to the pdf entitled *DanKuoyuFgf200128withChanges.pdf*). One thought on the latter might be inability of exogenous MRF to open the endogenous MRF loci in the absence of Fgf-driven high Tbx activity. But we have no data or obvious route to addressing these issues without a germline transgenic way to trigger MRF induction.

To address which FGF ligands mediate the activation MRF activation, the authors have resorted to morpholino oligonucleotide mediated knock down of individual FGFs. The use of this approach, with all its caveats, is somewhat disappointing given the well-established methods for targeted gene inactivation in the zebrafish.
Based on these data, the authors conclude that the combined activity of two FGFs (4 and 8a) is required for the activation of myod and myf5.
The authors next investigated the role of Tbx transcription factors as mediators of the MRF gene regulation by FGF. They show that inhibition of FGF signaling abolishes tbxta and tbx16 expression in trunk mesoderm and, based on morpholino knockdown experiments that Tbx16 is required for the activation of myod in preadaxial cells. This conclusion is supported by the finding that injection of FGF4 mRNA does not induce myod or myf5 expression in tbx16 mutant embryos, whereas injection of tbx16 mRNA can induce both myod and myf5 expression in FGF inhibitor (SU5402) treated embryos.
Taken together, these data provide reasonable support for the authors' second conclusion (line 444) that Fgf acts through Tbx16 to drive initial myogenic events in the adaxial cell lineage.
Evidence that TBX transcription factors are direct regulators of MRF genes is based on ChIPseq analysis. These reveal peaks that overlap with histone methylation peaks upstream of the myf5 and myoD genes. Whilst these data are consistent with the conclusion that Tbx16 activates transcription of myf5 and myod by binding to the elements thus identified, they do not constitute definitive proof of such a role. Mutation of these elements would provide a test of this conclusion.

This point is formally correct. However, we believe that making a series of small mutations to disrupt the several Tbx binding sites in each gene is beyond what it is reasonable to request in the current manuscript.

© 2020. Published by The Company of Biologists under the terms of the Creative Commons Attribution License (https://creativecommons.org/licenses/by/4.0/).

In summary, this study provides answers to some unresolved issues concerning the regulation of MRF expression and skeletal muscle development in the zebrafish embryo and supports some interesting evolutionary speculation.

We are pleased the Reviewer finds the work convincing and the insights gained interesting.

Reviewer 2 Comments for the Author:
• Figure 1a. I would avoid using “con” as an abbreviation for control in this context as it is also the symbol for the hedgehog pathway mutation “chameleon”

We have changed ‘con’ to ‘control’ throughout the manuscript.

• Line 161. The comment about the effect of loss of shha on trunk versus tail muscle is a bit elliptic. As the authors themselves show (Fig S2), complete loss of HH activity results in loss of muscle from the trunk as well as the tail - so their point (I think) is that the tail is more sensitive to a reduction in Hh activity. This could be made more explicit.

The Reviewer is correct. We have changed the text along the suggested lines at ln 181-182.

• It would be good to have a control for the specificity of the effects of the Fgf morpholinos – eg. aplnrb expression

We have now addressed this issue experimentally. The new data is included in Fig. S3E, ln 222-3.

• In the cycloheximide experiments, explain how the levels of myoD expression were quantified (line 361)

This was simply assessed by ISH staining intensity. We now mention this and further clarified the text (at ln 390- 392).

• The manuscript is quite long and not always an easy read - I appreciate that it describes a complex process but I think some re-writing could improve clarity and make it more accessible to readers who are not totally immersed in the subject.

We have shortened the manuscript and tried to enhance our main messages.

Reviewer 3 Advance Summary and Potential Significance to Field:
This manuscript is a tour de force analysis of slow-twitch muscle specification during trunk versus tail development. The data are of high quality and reported appropriately. The work shows that FGF and Tbx 16 cooperate to induce myogenic regulatory factor expression (ChIP- deq data show Tbx16 directly activates myogenic regulatory factors), providing a novel link between mesoderm induction and subsequent specification of muscle. Taken together, this manuscript fills an important hole in our understanding of muscle development.

We thank the Reviewer for her/his enthusiasm.

Reviewer 3 Comments for the Author:
Major Comments: My major comments all have to do with the writing/presentation of the manuscript.
The manuscript is quite erudite - which in itself is not bad but I suspect there is no chance that a typical graduate student would actually make it through the manuscript and that would be a shame because there is beautiful developmental biology in this manuscript.

Some suggestions to help with clarity (it took this reviewer an hour to get through a first read):
1. In the introduction it is not clear when the authors are discussing commonalities between mice and fish and when they are just referencing fish work - please make this more explicit. Also, it might be useful to including frogs/chickens as appropriate in the Tbx discussion.

© 2020. Published by The Company of Biologists under the terms of the Creative Commons Attribution License (https://creativecommons.org/licenses/by/4.0/).

We have gone through the introduction and clarified at all points precisely what kinds of vertebrate were studied in the quoted papers.

2. There are so many genes tested and tools used/different timing of treatments/analysis that it is a slog to get through the manuscript. Suggestions - please write that treating at 30% epiboly in the first results paragraph,

Done, and in legend.

please define genes/inhibitors as you use them,

Done.

and it would be fantastic if the authors could use cartoons throughout the manuscript to highlight their design, rationale, and results.

Many of the experiments (Figs 1-4, S1,S4,S5) involve either a simple genetic cross or treatment with a single drug/reagent mix and then analysis at a defined time, yielding two outcome conditions (control and experiment). Other experiments involve a simple heterozygous in-cross combined with RNA or morpholino injection (Figs 4,5,7,8, S2,S3), yielding four outcomes (mutant or sib control ± treatment). These facts are written on each panel and stated in the relevant legend and do not merit additional diagrams. Where the experiment is more complex (e.g. Fig. 6E) we include a diagram (Fig. 6D).

We now also include a summary schematic of our resulting model (Fig. S7) and a Table of the outcomes of experiments (Table S5).

3. While the discussion about potential evolutionary implications is interesting, I have some concerns. One, I'm not convinced that it is appropriate to state in the abstract that the findings provide insight into ancestral vertebrate trunk myogenesis given how derived zebrafish are... and one would maybe need to do comparative studies in sharks? I would suggest that the authors consider focusing less on evolution in the introduction - where there are some broad statements that are slightly disingenuous (for example, "how might deuterostome muscle have formed prior to evolution of the notochord" - this was confusing to me because invertebrates are perfectly good at generating muscle.... and at least some of the mechanisms appear to be conserved.....). The discussion seems to be a more appropriate place for speculation about evolution of muscle development.

While the Reviewer is correct that all statements about evolution are speculative, we believe our Introduction positions the paper to focus on the key aspects of our findings. Despite the relatively derived position of zebrafish within bony fish, with respect to trunk and tail segmentation zebrafish are no more 'derived' than a mouse or human and we believe these species are all well-suited to phylogenetic comparisons across the vertebrata. The synapomorphies (shared-derived characteristics) in Tbx/Fgf and Hh function that we describe between mice and zebrafish are clearly parsimoniously explained by a common ancestral condition, as opposed to the alternative hypothesis of convergent evolution. We believe the question of how muscle formed in ancestral deuterostomes before the notochord arose is important, and that our findings raise a significant hypothesis: that it was through Fgf-driven activation of Tbx6/Tbxt gene function. It would, of course, be nice to test our hypothesis by doing similar experiments in amphioxus, lampreys/hagfish, sharks, or coelocanths. However, such manipulations in these species are not possible for us and go beyond what is reasonable in the current manuscript. We prefer to retain the current focus as we believe this is a major contribution of our experimental study and its associated thinking. If we are wrong, and future studies in more basal chordates fail to support the view that Fgf/Tbx is the ancestral route to deuterostome trunk myogenesis, then those studies will be able to quote our paper in their Introductions, before trashing it, and science will advance. We have modified the Abstract to make it clear that we are suggesting a hypothesis, rather than yielding a definitive conclusion, on how trunk muscle was patterned in the ancestral vertebrate.

© 2020. Published by The Company of Biologists under the terms of the Creative Commons Attribution License (https://creativecommons.org/licenses/by/4.0/).

Minor comments:
-The Fgf4 expression is fairly drastic, do the authors have the ability to quickly do more mosaic expression or genetic mosaic analysis to determine whether clusters of Fgf4 expressing cells are sufficient to initiate MRF expression?

This is a good suggestion and we were hopeful that such localised signalling would provide insight into which cell types were sensitive and how far the Fgf signal could travel. We injected Fgf4 RNA into one cell at 32 cell stage and found patchy induction of *myf5* that locates near the Fgf4 source on some occasions, but gives no very clear-cut and consistent local effect (see Reviewer Fig. 1 below). We are not sure why this is the result. We tried triple staining for Tbxt to check whether the location of over-expressing cells mattered, but could make no more sense of the data. Despite our efforts to control variables, we suspect a combination of Fgf4 level, variable clonal expansion of the injected cell, and precise location to ectoderm or mesendoderm may all affect the outcome. We do not think the data we have been able to obtain adds functional insight to the manuscript and have therefore not included it in the revised version.

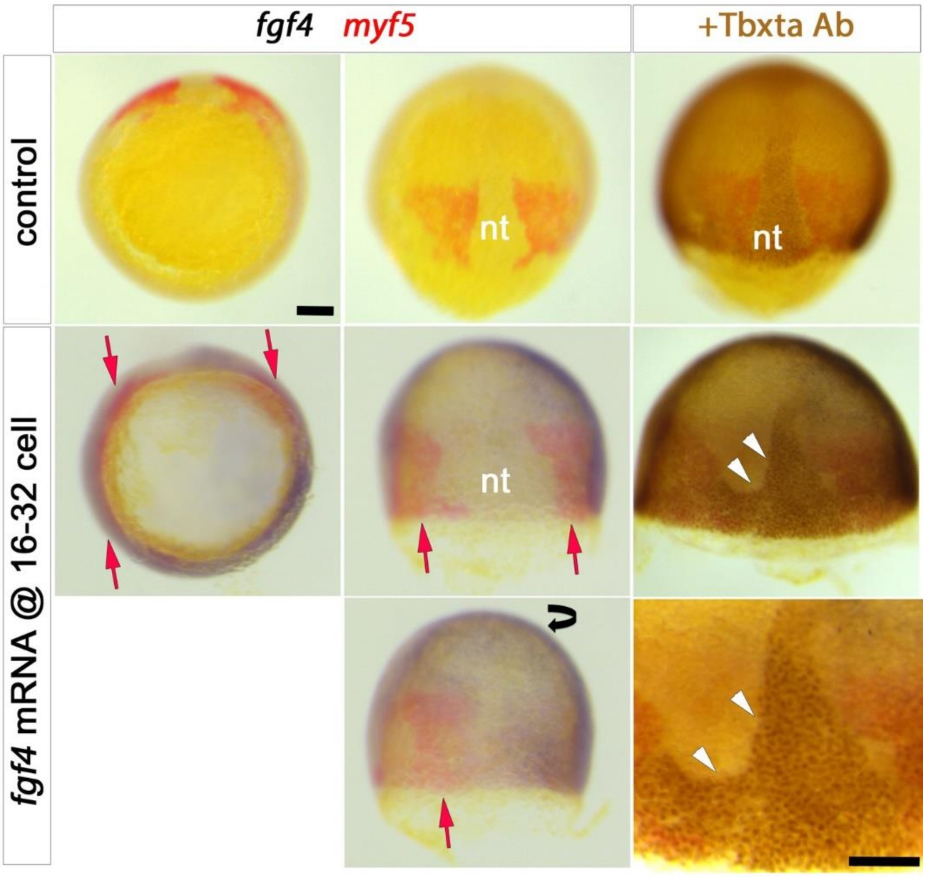

Reviewer Figure 1. Mosaic expression of *fgf4*.
Double ISH at 80% epiboly of embryos injected with *fgf4* RNA into one cell at 16-32 cell stage showing exogenous *fgf4* RNA (blue) and *myf5* mRNA (red). Embryos were subsequently immunostained for Tbxt (anti-Ntl) antibody (white arrowheads, brown, right column).
Top row shows an uninjected embryo. Below, an example of injected embryo shown in vegetal (left), dorsal (centre) and dorsal flattened (right, with Tbxt, magnified below), and lateral (centre bottom). Red arrows indicate ectopic *myf5* expression. nt, notochord. Bars = 100 µm.

© 2020. Published by The Company of Biologists under the terms of the Creative Commons Attribution License (https://creativecommons.org/licenses/by/4.0/).

Second decision letter

MS ID#: DEVELOP/2019/184689

MS TITLE: Fgf-driven Tbx protein activities directly induce myf5 and myod to initiate zebrafish myogenesis

AUTHORS: Daniel P.S. Osborn, Kuoyu Li, Stephen J Cutty, Andrew C Nelson, Fiona C Wardle, Yaniv Hinits, and Simon M Hughes
ARTICLE TYPE: Research Article

I am happy to tell you that your manuscript has been accepted for publication in Development, pending our standard ethics checks. The referee reports on this version are appended below.

Reviewer 1

Advance summary and potential significance to field

The authors have addressed all my previous concerns, including adding new experimental data.

Comments for the author

This nice study makes a number of important insights into the onset of vertebrate myogenesis.

Reviewer 3

Advance summary and potential significance to field

The authors have done a great job of addressing reviewer concerns and this is a very nice paper!

Comments for the author

The authors have done a great job of addressing reviewer concerns and this is a very nice paper!

© 2020. Published by The Company of Biologists under the terms of the Creative Commons Attribution License (https://creativecommons.org/licenses/by/4.0/).
